# Safety and Immunogenicity Study of a Bivalent Vaccine for Combined Prophylaxis of COVID-19 and Influenza in Non-Human Primates

**DOI:** 10.3390/vaccines12101099

**Published:** 2024-09-26

**Authors:** Ekaterina Stepanova, Irina Isakova-Sivak, Victoria Matyushenko, Daria Mezhenskaya, Igor Kudryavtsev, Arina Kostromitina, Anna Chistiakova, Alexandra Rak, Ekaterina Bazhenova, Polina Prokopenko, Tatiana Kotomina, Svetlana Donina, Vlada Novitskaya, Konstantin Sivak, Dzhina Karal-Ogly, Larisa Rudenko

**Affiliations:** 1 Institute of Experimental Medicine, Saint-Petersburg 197022, Russia; isakova.sivak@iemspb.ru (I.I.-S.); matyshenko@iemspb.ru (V.M.); dasmez@iemspb.ru (D.M.); kudryavtsev.iv@iemspb.ru (I.K.); arina8goshina@gmail.com (A.K.); anna.k.chistiakova@gmail.com (A.C.); rak.ay@iemspb.ru (A.R.); pi.prokopenko@gmail.com (P.P.); kotomina@iemspb.ru (T.K.); novitskaya.vv@iemspb.ru (V.N.); rudenko.lg@iemspb.ru (L.R.); 2 Smorodintsev Research Institute of Influenza, Saint-Petersburg 197376, Russia; konstantin.sivak@influenza.spb.ru; 3 Center of Preclinical Research, Research Institute of Medical Primatology, Sochi 354376, Russia; karal_5@mail.ru

**Keywords:** SARS-CoV-2, influenza, bivalent vaccine, virus-vectored vaccine, non-human primates, rhesus monkeys, preclinical study

## Abstract

Background. Influenza and SARS-CoV-2 viruses are two highly variable pathogens. We have developed a candidate bivalent live vaccine based on the strain of licensed A/Leningrad/17-based cold-adapted live attenuated influenza vaccine (LAIV) of H3N2 subtype, which expressed SARS-CoV-2 immunogenic T-cell epitopes. A cassette encoding fragments of S and N proteins of SARS-CoV-2 was inserted into the influenza NA gene using the P2A autocleavage site. In this study, we present the results of preclinical evaluation of the developed bivalent vaccine in a non-human primate model. Methods. Rhesus macaques (*Macaca mulatta*) (n = 3 per group) were immunized intranasally with 7.5 lg EID_50_ of the LAIV/CoV-2 bivalent vaccine, a control non-modified H3N2 LAIV or a placebo (chorioallantoic fluid) using a sprayer device, twice, with a 28-day interval. The blood samples were collected at days 0, 3, 28 and 35 for hematological and biochemical assessment. Safety was also assessed by monitoring body weight, body temperature and clinical signs of the disease. Immune responses to influenza virus were assessed both by determining serum antibody titers in hemagglutination inhibition assay, microneutralization assay and IgG ELISA. T-cell responses were measured both to influenza and SARS-CoV-2 antigens using ELISPOT and flow cytometry. Three weeks after the second immunization, animals were challenged with 10^5^ PFU of Delta SARS-CoV-2. The body temperature, weight and challenge virus shedding were monitored for 5 days post-challenge. In addition, virus titers in various organs and histopathology were evaluated on day 6 after SARS-CoV-2 infection. Results. There was no toxic effect of the immunizations on the hematological and coagulation hemostasis of animals. No difference in the dynamics of the average weight and thermometry results were found between the groups of animals. Both LAIV and LAIV/CoV-2 variants poorly replicated in the upper respiratory tract of rhesus macaques. Nevertheless, despite this low level of virus shedding, influenza-specific serum IgG responses were detected in the group of monkeys immunized with the LAIV/CoV-2 bivalent but not in the LAIV group. Furthermore, T-cell responses to both influenza and SARS-CoV-2 viruses were detected in the LAIV/CoV-2 vaccine group only. The animals were generally resistant to SARS-CoV-2 challenge, with minimal virus shedding in the placebo and LAIV groups. Histopathological changes in vaccinated animals were decreased compared to the PBS group, suggesting a protective effect of the chimeric vaccine candidate. Conclusions. The candidate bivalent vaccine was safe and immunogenic for non-human primates and warrants its further evaluation in clinical trials.

## 1. Introduction

The SARS-CoV-2 and influenza viruses are two highly variable pathogens that cause millions of illnesses and can co-infect individuals. It was demonstrated in different studies that a robust virus-specific T-cell response is correlated with a mild or asymptomatic course of COVID-19 [1,2]. The role of tissue-resident memory cells was demonstrated in experimental models [3]. It is therefore reasonable to suggest that the formation of virus-specific tissue resident memory T cells in respiratory organs will provide protection against severe COVID-19 disease. Markov et al. [4] reported that patients who had cells specific for S and/or N epitopes in their lungs had a more favorable outcome of COVID-19 than patients with cells specific for ORF1ab epitopes. For SARS virus, the investigation of linear epitopes in rhesus macaques revealed the development of antibody-dependent enhancement of viral infection associated with a single epitope in the spike protein [5]. Thus, targeted stimulation of T-cell immunity by antigens with engineered epitope composition is a promising strategy for the development of next-generation vaccines.

A variety of vaccines utilizing this strategy are currently under development. Preclinical studies of vectored polyepitope vaccines based on AAV-vector [6], lentivirus vector [7] and vaccinia virus [8] have recently been published. In addition, polyepitope constructs targeting conserved T-cell epitopes of S, N, M and ORF1ab proteins are used as components of the complex antigen in the BioNTech mRNA BNT162b4 vaccine [9], vaccinia virus-based SARS-CoV-2 vaccine [10] and UB-612 vaccine [11,12,13]. Another peptide COVID-19 vaccine has been studied in trials as a T-cell stimulator in people with B-cell deficiency [14].

Influenza virus is a highly variable pathogen that requires annual vaccination to keep the population’s immune system up to date. The idea of combining prophylaxis against two respiratory pathogens in a single vaccine has led to the development of a number of bivalent vaccine formulations. Several vaccines for combined prophylaxis of COVID-19 and influenza are based on influenza vectors, most of them targeting RBD expression [15,16,17,18,19,20]. We previously developed a panel of candidate bivalent vaccines for combined prophylaxis of SARS-CoV-2 and influenza virus based on the live attenuated influenza vaccine (LAIV) strain [21]. The described constructs were aimed at stimulating T-cell immunity and were studied in vitro on human peripheral blood mononuclear cells (PBMCs) and in experiments on mice and hamsters. Based on our results, we selected the best candidate that stimulated immunity against influenza and SARS-CoV-2 and provided partial protection against SARS-CoV-2 in the hamster challenge study. The SARS-CoV-2 cassette in this most promising candidate was inserted into the NA gene and encoded SARS-CoV-2 spike and nucleoprotein fragments [21].

Rhesus macaques are a common model for preclinical studies of human vaccines and therapeutics. Various subtypes of influenza viruses have been studied in this model [22,23,24,25,26,27,28,29,30]. *Macaca mulatta* has also been shown to be a suitable primate for modeling SARS-CoV-2 infection [31,32,33,34] (reviewed in [35]); therefore, preclinical studies of a number of COVID-19 vaccines have been conducted in this animal model [36,37,38].

In this work, we studied a candidate bivalent live vaccine developed on the basis of a licensed H3N2 LAIV strain with insertion of a previously selected SARS-CoV-2 T-cell cassette [21] into the NA segment in a rhesus macaque model. Primates were intranasally immunized with the investigated vaccines and monitored for their health status. Five weeks after the second immunization, the animals were intranasally inoculated with a dose of SARS-CoV-2 to assess the protective effect of vaccination and to prove the absence of a vaccine-associated disease enhancement effect, since there is evidence that SARS-CoV vaccines can induce antibody-dependent disease enhancement (ADE) following coronavirus infection [5,39,40,41,42].

## 2. Materials and Methods

### 2.1. Viruses, Cells and Proteins

#### 2.1.1. Viruses

A/Brisbane/34/2018 (H3N2) influenza virus was obtained from a WHO collaborating center (Melbourne, Australia). For this study, two strains were rescued on the basis of cold-adapted master donor virus for LAIV A/Leningrad/134/17/57 (H2N2) and A/Brisbane/34/2018 (H3N2) strains, and both inherited six genes from Len/17 and HA from A/Brisbane/34/2018. The experimental bivalent vaccine strain, FluCoVac-96, expressed modified NA gene of A/Brisbane/34/2018, which encoded the SARS-CoV-2 T-cell cassette described in detail in our previous work [21]. In brief, the cassette encodes combined fragments of nucleoprotein and spike proteins of SARS-CoV-2 (Wuhan lineage) virus, and the full sequence of the cassette is published in [21]. The control H3N2 LAIV strain had the unmodified NA of A/Brisbane/34/2018 strain.

Influenza viruses were propagated in 10–11-day-old embryonated chicken eggs at 33 °C, aliquoted and stored at −70 °C. For immunological assays, egg-propagated H3N2 LAIV virus was purified in a sucrose gradient as described in [43].

The SARS-CoV-2 strain hCoV-19/Russia/SP48-1339/2021 (Delta lineage) propagated in Vero C1008 cells was used in a monkey challenge experiment. All operations with SARS-CoV-2 viruses and virus-containing materials were conducted in a BSL-3 facility by certified personnel.

#### 2.1.2. Cells

MDCK cell line (ATCC CCL-34, canine kidney cell culture) was used for assessment of influenza growth characteristics. Vero E6 cell culture (ATCC C1008, African green monkey cells) was used for SARS-CoV-2 growth. Cells were maintained according to standard protocols in Dulbecco’s Modified Eagle Medium (DMEM) (Paneco, Moscow, Russia) with 10% fetal bovine serum (FBS) (Capricorn, Ebsdorfergrund, Germany) and 1 × antibiotic–antimycotic solution (Capricorn, Ebsdorfergrund, Germany) at 37 °C and 5% CO_2_.

#### 2.1.3. Proteins and Peptides

The recombinant SARS-CoV-2 (Wuhan lineage) N protein was expressed in *E. coli* BL21 (DE3) strain, purified from the sonicated biomass using immobilized metal affinity chromatography (IMAC) and dialyzed against PBS [44]. Proteins were stored at −70 °C in aliquots.

### 2.2. In Vitro Studies of the Experimental Vaccine Virus

#### 2.2.1. Replication in Eggs and MDCK Cells

Influenza virus titers were estimated by limiting dilutions in 10–11-day-old developing chicken embryos at 33 °C for 72 h. Viral titers in MDCK cells were assessed either by limiting dilutions on 96-well plates or by plaque assay on 6-well plates. Cells infected with virus dilutions were incubated at 33 °C for 72 h in DMEM supplemented with 1 × AA and 1 µg/mL of TPCK trypsin. The titers were calculated according to the Reed and Muench method [45] and expressed as lgEID_50_/mL, lgTCID_50_/mL or lgPFU/mL.

#### 2.2.2. Genetic Stability of Experimental Vaccine Virus

The genetic stability assessment of FluCoVac-96 recombinant vaccine was conducted after 5 and 10 sequential passages in eggs. For RNA extraction, Biolabmix column kit (Biolabmix, Novosibirsk, Russia) was used. To amplify the genome fragments, specific primer sets with One-Step RT-PCR kit (Biolabmix, Novosibirsk, Russia) were used. After the standard procedure of DNA extraction from 1% agarose gel with Evrogen S-cup column kit (Evrogen, Moscow, Russia), the sequencing reaction was performed using BigDye™ Terminator v3.1 Cycle Sequencing Kit (Thermofisher Scientific, Waltham, MA, USA). Sanger sequencing was performed using 3130 xl Genetic Analyzer (Applied Biosystems, USA) with subsequent analysis of the data with Lasergene 7.1 software.

### 2.3. Experimental Manipulations on Rhesus Macaques

Animal experiments were performed according to the Directive 2010/63/EU [46]. Animal study design was approved by local ethics committee of Research Institute of Medical Primatology (protocol №101 dated 31 October 2022).

Nine male rhesus macaques (*Macaca mulatta*), 3–4 years old and weighing 3–5 kg, were obtained from the Federal Budgetary Scientific Institution of Medical Primatology (Sochi, Russian Federation). Before the start of the studies, the animals were quarantined for 2 weeks. The presence of antibodies to influenza and SARS-CoV-2 was assessed before the start of the experiment. During the experiment, the animals were kept in individual cages in rooms with temperature 23–26 °C, relative humidity 55 ± 15% and a 12/12 h light/dark cycle. The diet of animals was balanced according to species preferences; the animals had free access to water.

#### 2.3.1. Immunization and Sample Collection

**Figure 1:**
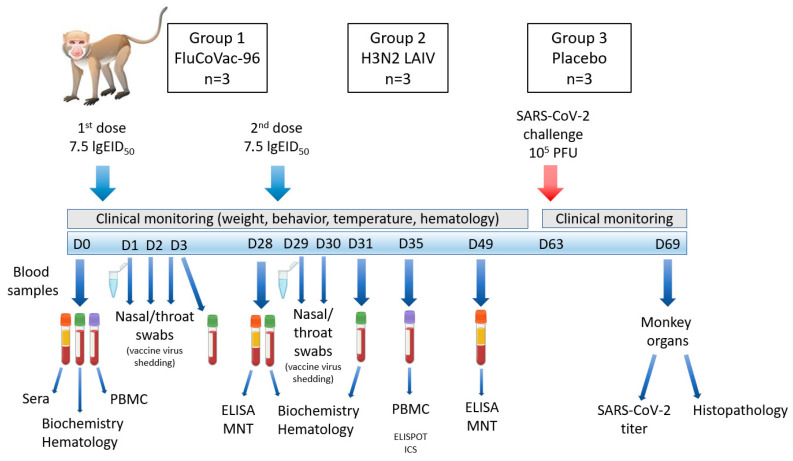
The scheme of the experiment on assessment of safety and immunogenicity of the FluCoVac-96 in rhesus monkeys. D—days of the experiment; PBMCs—peripheral blood mononuclear cells; MNT—virus microneutralization test; ICS—intracellular cytokine staining.

Animals (3 per group) were intranasally immunized with 7.5 lg EID_50_ of the studied influenza viruses, or with chorioallantoic fluid as a placebo, in a volume of 500 μL using a dispenser sprayer. The immunizations were performed twice within a 4-week interval. Clinical assessment of animals was performed throughout the study, and blood samples were collected on days 0, 3, 28, 31, 35 and 49 for hematological/biochemical and immunological analyses (Figure 1). Nasal and throat swabs were taken on days 1–3 after the 1st vaccine dose and on days 1–2 after the booster dose. Synthetic fiber swabs were used for these procedures, and the tips of the swabs were placed in individual tubes with 1 mL of sterile PBS and were stored at −70 °C. Peripheral blood mononuclear cells (PBMCs) of monkeys were isolated from peripheral blood sampled in vacutainer tubes with EDTA on days 0 and 35 of the experiment. PBMCs freshly isolated at day 35 were used for ELISPOT, while cells collected at both day 0 and day 35 were cryopreserved in 95% HyClone FBS + 5% DMSO for subsequent flow cytometry analyses. Five weeks after the second dose, the animals were transferred to the facilities with BSL-3 levels and were infected with SARS-CoV-2 virus.

#### 2.3.2. Clinical Assessment

Evaluation of animal health status after immunization and SARS-CoV-2 challenge was performed by multiple parameters, such as appearance, skin and coat condition, eye condition, mucous membranes, nose/breathing character, stool character, appetite, body position, behavior and coordination of movements (the full list of the clinical parameters assessed is in Table S1). Body weight was monitored using an electronic scale (AND, Tokyo, Japan) on days 0, 7, 14, 21, 28, 35, 42 and 63–69. Body temperature was measured in the rectum using an electronic thermometer (OMRON, Kyoto, Japan) on days 0–7, 14, 21, 28–35, 42, 49 and 63–69.

Blood samples for hematological and biochemical analyses were taken before immunization (day 0) and then on days 3, 28 and 31 of the experiment. The following hematological parameters were measured: hemoglobin, erythrocyte count, color index, leukocyte count, leukocyte formula (lymphocytes, monocytes, neutrophils, eosinophils, basophils), platelet count, hematocrit, average cell volume (erythrocytes), mean corpuscular volume, mean corpuscular hemoglobin and mean corpuscular hemoglobin concentration. Measurements were performed on an automatic hematology analyzer MEK-7300K (Celltac ES, Tokyo, Japan). The following biochemical parameters were measured: sodium, creatinine, lactate dehydrogenase, alkaline phosphatase, total bilirubin, total protein, urea, glucose, triglycerides, aspartate aminotransferase, alanine aminotransferase, total cholesterol and potassium. Measurements were performed on a BioLit-8020 automatic biochemical analyzer (URIT Medical Electronic Group, Guilin, China). Coagulometry was performed on TS 4000 Plus (High Technology Inc., North Attleborough, MA, USA), where activated partial thromboplastin time (aPTT), prothrombin time and fibrinogen were measured.

#### 2.3.3. Experimental Challenge with SARS-CoV-2

On day 63 of this study, monkeys were transferred to the facilities with BSL-3 levels and were infected with SARS-CoV-2 virus at a dose of 10^5^ PFU by intranasal inoculation. Clinical examination of the infected animals, as well as nasal and throat swabs collection for virology studies, were performed daily after the challenge. On day 69 of the experiment, the animals were humanely euthanized, and their organs were inspected for SARS-CoV-2. The swab material was used for RNA extraction with M-sorb-OOM (Syntol, Moscow, Russia) kit, and real-time PCR with reverse transcription was performed using PCR-RT-2019-nCoV kit and AHK-32M PCR machine (Syntol, Moscow, Russia). In addition, the swab material was used for live virus PFU assessment in Vero cell culture. On day 69 (6 days after SARS-CoV-2 challenge), the animals were humanely euthanized, and the lungs, trachea, heart, kidneys, liver and spleen fragments were removed for examination. Lung fragments were mechanically homogenized, and the titer of SARS-CoV-2 virus in the suspension was assessed by virus PFU formation in Vero E6 cell culture, as described above.

All operations with SARS-CoV-2 viruses, infected animals and virus-containing materials were conducted in a BSL-3 facility by certified personnel.

### 2.4. Assessment of Vaccine Virus Shedding

To detect replication of influenza vaccine virus, swab material was inoculated into developing chicken embryos in serial dilutions, and a portion of the swab material was taken for RNA isolation for subsequent endpoint PCR and real-time PCR tests. Eggs were incubated for 72 h at 33 °C, and HA reaction with chicken red blood cells was performed for virus identification. Viral RNA was purified with AmpliPrime RIBO-prep precipitation kit (Amplisens, Moscow, Russia). Before the PCR step, the samples were treated with DNAse RQ1 (Promega, Madison, WI, USA), and then real-time PCR with reverse transcription was performed using QuantStudio 1 machine (Thermofisher Scientific, Waltham, MA, USA) and Biolabmix one-step reverse transcription real-time PCR kit (Biolabmix, Novosibirsk, Russia). The protocol included the following steps: 30′ 50 °C, 2′ 95 °C, 45 cycles (95 °C 15″; 55 °C 30″ with fluorescence registration). The samples were processed in duplicates. The following oligonucleotides and FAM-labeled probe specific to influenza A M gene were used: 5′-GACCAATCCTGTCACCTCTGAC-3′; 5′-AGGGCATTTTGGACAAAGCGTCTA-3′; 5′-/6-FAM/-TGCAGTCCTCGCTCACTGGGCACG-/BHQ-1/-3′ ([47] with modifications). Ten-fold dilutions of the viral RNA extracted from the egg-derived virus were used for standard curve plotting. The data were analyzed with QuantStudio Design and Analysis Software v1.5.1.

The endpoint RT-PCR was performed with universal oligonucleotides to influenza A genes according to previously described protocol [48]. The PB2 and NA segment fragments were amplified with universal oligonucleotides, and positive samples were subjected to Sanger sequencing to verify the etiology of the infection.

### 2.5. Assessment of Humoral Immune Responses

#### 2.5.1. Hemagglutination Inhibition (HAI) Assay

HAI assay was performed using standard procedures described in WHO Manual on Animal Influenza Diagnosis and Surveillance [49]. Briefly, serum samples were treated with receptor-destroying enzyme (RDE) and serially 2-fold diluted in PBS starting from 1:10, in a volume of 50 µL, in duplicates. Four HA units of the H3N2 LAIV virus were added to each well at an equal volume, and after 30 min of incubation at RT, 100 µL of 0.5% chicken red blood cells were added, and the plates were read 30 min later.

#### 2.5.2. ELISA

The levels of influenza-specific serum IgG antibodies were assessed by ELISA. Briefly, 96-well high-binding polystyrene plates (Thermofisher Scientific, Waltham, MA, USA) were coated with sucrose gradient-purified H3N2 LAIV virus (16 HA units per well) in carbonate–bicarbonate buffer (pH 7.4) for 20 h at +4 °C. After washing and blocking with BSA, 2-fold serum dilutions were added in duplicates for 1 h at 37 °C. For IgG detection, Goat Anti-Monkey IgG HRP-conjugated antibodies (ab112767, Abcam, Cambridge, UK) were used according to a standard protocol with a 1-Step TMB Substrate Solution (Thermofisher Scientific, Waltham, MA, USA). The results were read at 450 nm (OD_450_) using xMark Microplate Spectrophotometer (BioRad, Hercules, CA, USA). The endpoint titer was defined as the serum dilution that gave an optical density at least two times higher than that of the control wells (virus without serum).

SARS-CoV-2-specific serum IgG antibodies were measured against recombinant N protein expressed in bacterial cells (100 ng/well) [44] using procedures described above.

#### 2.5.3. Microneutralization Assay

For virus neutralization assessment, the sera were treated with receptor-destroying enzyme (RDE, Denka Seiken, Tokyo, Japan) for unspecific inhibitors removal. Two-fold dilutions of serum samples were mixed with 100 TCID_50_ of A/Brisbane/34/2018 (H3N2) virus per each well, incubated for 1 h and then transferred to 96-well plates with confluent monolayers of MDCK cells. The mixtures were incubated at 37 °C, 5% CO_2_ for 1 h and then the inoculum was removed, culture medium containing corresponding serum dilutions was added to appropriate wells and the plates were incubated for 48 h at 37 °C, 5% CO_2_. To detect the virus neutralization effect, after 48 h medium was removed and cells were fixed (80% acetone solution). Virus replication in wells was detected using ELISA protocol with HRP-conjugated anti-influenza NP monoclonal antibody (OOO “PPDP”, St. Petersburg, Russia). The 50% inhibitory concentration (IC_50_) was calculated with a four-parametric nonlinear regression method using GraphPad Prism 10.1.

### 2.6. Assessment of Cell-Mediated Immune Responses

#### 2.6.1. ELISPOT

Virus-specific interferon-gamma (IFNγ)-secreting cells were counted in PBMCs of immunized monkeys on day 35 (7 days after the booster dose) of the experiment. The cells were isolated with Ficoll gradient (described in [43]), and live cells were counted with TC-20 automatic cell counter (BioRad, Hercules, CA, USA). The 500 000 cells in 100 µL/well were stimulated with sucrose-purified influenza virus (2 MOI) or recombinant SARS-CoV-2 N protein (2 µg/mL). The mixtures were added to the plate of FluoroSpot Plus: Human IFN-γ/TNF-α (Mabtech, Hamburg, Germany) kit and incubated for 18 h at 37 °C, 5% CO_2_. Detection of spots was performed according to the manufacturer’s protocol. Spots were counted with AID vSpot Spectrum (Advanced Imaging Devices GmbH, Strassberg, Germany).

#### 2.6.2. Intracellular Cytokine Staining (ICS)

Cryopreserved monkey PBMCs collected at day 0 and 35 were used for intracellular cytokine staining with flow cytometry analysis. Cells were gently thawed and then washed in RPMI medium, counted and added to 96-well plates in RPMI medium supplemented with 10% FBS and 1x antibiotic–antimycotic solution (all from Capricorn, Ebsdorfergrund, Germany). For each stimulation, 1–1.5 × 10^6^ cells were used. Cells were left for 5 h at 37 °C, 5% CO_2_ for normalization of physiological condition and then stimulated with sucrose-purified H3N2 LAIV virus (2 MOI) or recombinant SARS-CoV-2 N protein (2 µg/mL) for 16 h at 37 °C, 5% CO_2_. Unstimulated cell samples were also prepared for each animal. The protein transport inhibitor BD Golgi Plug (BD Biosciences, Franklin Lakes, New Jersey, USA) was then added to each well and incubated for another 5 h. After incubation, cells were stained with the following antibodies cross-reactive with rhesus macaque molecules: ZombieAqua (live/dead cells) (Biolegend, San Diego, CA, USA), AF700-CD8 (SK1 clone) (344724, Biolegend, San Diego, CA, USA), APC-CD4 (OKT4 clone) (317416, Biolegend, San Diego, CA, USA), CD45RA-ECD (2H4LDH11LDB9, 2H4 clone) (Beckman Coulter, Brea, CA, USA) and PerCP-Cy5.5 CD197 (G043H7 clone) (353220, Biolegend, San Diego, CA, USA). Cells were then fixed and permeabilized using BD Cytofix/Cytoperm™ Fixation/Permeabilization Kit (BD Biosciences, Franklin Lakes, NJ, USA), and cytokines were stained with the following antibodies: AF488-IFNγ (4S.B3 clone) (502515, Biolegend, San Diego, CA, USA), BV421-TNFα (MAb11 clone) (502932, Biolegend, San Diego, CA, USA) and PE-IL-2 (MQ1-17H12 clone) (500307, Biolegend, San Diego, CA, USA). The cross-reactive clones of the antibodies were selected using information from the manufacturer, Reactivity Database on NHP resource [50]. The clones we selected were used for immunophenotyping in several studies on rhesus monkeys [51,52,53]. Flow cytometry was performed on a Navios flow cytometer (Beckman Coulter, Brea, CA, USA), and data were analyzed using Kaluza Analysis software v 2.1 (Beckman Coulter, Brea, CA, USA).

### 2.7. Histopathological Analysis

Fragments of lungs, trachea, heart, kidneys, liver and spleen collected on day 6 after SARS-CoV-2 challenge infection (approximately 1 sm^3^ in size) were carefully excised and placed in tubes with 10% formaldehyde (Formalin solution, 10% histological tissue fixative; SIGMA-Aldrich, St. Louis, MI, USA). The samples were subjected to histopathological examination as previously described [21]. In brief, the tissues were embedded in paraffin by Tissue-Tek VP1 station (Sakura Finetek, Tokyo, Japan), and histological sections (l–3 μm) were prepared and stained with hematoxylin and eosin, with additional alcian blue staining (to detect acid glycosaminoglycans, mucus and cartilage). Histological preparations were studied using light optical microscopy on a Leika DM 1000 microscope (Leica Microsystems, Wetzlar, Germany) at magnifications of 40–1000×. Representative figures show micrographs at 40× and 200× total magnification. Microphotographs were prepared using the ADF Pro program.

### 2.8. Statistical Analyses

The results were analyzed using GraphPad Prism 10 software. The exact statistical methods are indicated in appropriate tables and figures. The differences were considered significant at *p* < 0.05.

## 3. Results

### 3.1. In Vitro Characterization of the Recombinant LAIV/CoV-2 Virus (FluCoVac-96)

**Figure 2:**
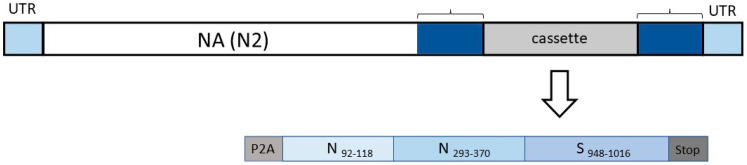
The scheme of modified NA segment of the FluCoVac-96 and the SARS-CoV-2 cassette insertion. UTR—untranslated regions of influenza NA segment; P2A—sequence encoding self-cleaving site of porcine teschovirus-1; N, S—SARS-CoV-2 proteins, the amino acid residues are listed in subscript; Stop—3 stop-codons at the end of the cassette. The additional fragment of NA sequence is shown in blue.

A recombinant influenza virus, FluCoVac-96, was rescued using six genes of A/Leningrad/17 LAIV master donor virus and intact HA and modified NA genes of the A/Brisbane/34/2018 (H3N2) influenza strain. NA modification was performed using the same protocol described previously [21]. The scheme of NA modification and cassette composition are shown in Figure 2. The cassette is inserted immediately after the NA ORF end (without a stop codon), and autonomous processing of the cassette is provided by a P2A self-cleavage site. The cassette ends with three stop codons, after which an additional NA sequence fragment is added to improve virus packaging. As a result, the NA protein is not modified in the virus particle, since the cassette is cleaved at the translation step.

**Figure 3:**
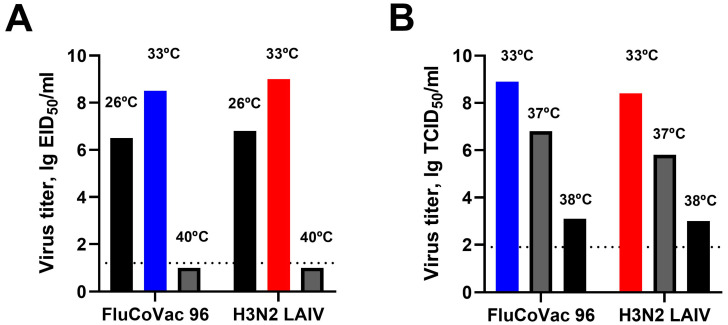
Replication of FluCoVac-96 and control H3N2 LAIV strain in chicken embryos and MDCK cells. (**A**) Reproduction in eggs at different temperatures; (**B**) reproduction in MDCK cells at different temperatures.

Replication of the chimeric influenza virus was evaluated in eggs and MDCK cells and compared with a control virus, a classical H3N2 LAIV reassortant strain derived from the same parental viruses. We assessed standard in vitro phenotypic markers that are used for LAIV quality control, growth of the viruses at optimum (33 °C) temperature, lowered (26 °C) temperature in eggs (phenotypic marker of cold adaptation), elevated temperature (40 °C in eggs, 38 °C in MDCK cells) and phenotypic marker of temperature sensitivity [54,55,56]. The strain was able to grow at 26 °C and did not replicate at 40 °C in chicken embryos, which is an in vitro marker of its safety (Figure 3B). Furthermore, the growth at 37–38 °C in MDCK cells was restricted, suggesting the inability of the vaccine viruses to grow in the lower respiratory tract of susceptible mammals. Importantly, the addition of the cassette to the NA segment did not affect the growth characteristics of the virus (Figure 3).

Genetic stability was assessed by Sanger sequencing after 5 and 10 egg passages. The strain maintained the cassette insertion after 5 and 10 passages, and there were no mutations in the inserted sequence, as well as in the entire modified NA segment. The presence of substitutions providing an attenuated phenotype of A/Leningrad/17-based strains was also confirmed after passaging [56].

### 3.2. Safety of the FluCoVac-96 Strain in Rhesus Monkeys

**Figure 4:**
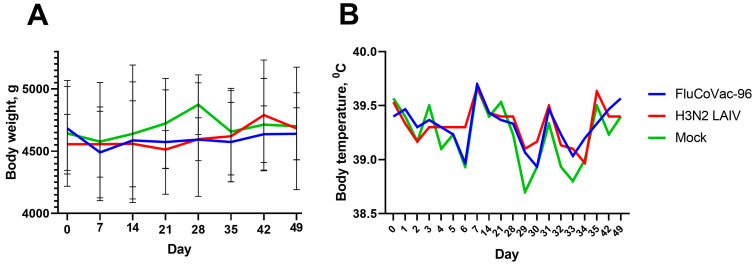
Body weight and temperature dynamics of the rhesus monkeys at D0-D49. Group mean values are presented. (**A**) Body weight dynamics (mean ± SD). (**B**) Body temperature dynamics. There were no significant differences in body weight and temperature dynamics between groups.

Nine male rhesus macaques, 2 to 7 years of age, were included in the experiment. Before the start of the experiment, the animals were examined for health status, and their weight and temperature were measured. For this preclinical study in monkeys, we selected a vaccine dose similar to the human dose of seasonal LAIV based on Len/17 MDV [57]. Licensed vaccines contain not less than 7 lg EID_50_ of each Flu A component [57,58], so to follow safety in this primate model, 7.5 lg EID_50_ was used. Three monkeys from each group received two doses of either FluCoVac-96 or H3N2 LAIV, 10^7.5^ EID_50_ each, four weeks apart. The control group was immunized with a placebo (allantoic fluid). The health status and clinical assessment of animals were monitored during the immunization phase (days 0–49), and no significant changes were observed in the clinical status of vaccinated animals, including food consumption and appetite. Several animals in all study groups showed a slight decrease in body weight from baseline values, which was most likely a consequence of stress (Figure 4A). Throughout the entire observation period, the body temperature of experimental animals of all groups remained at the level of normal values; changes in the average temperature of animals in the groups did not exceed 0.8 °C, and no significant differences between groups at all time points were detected (Figure 4B).

#### 3.2.1. Blood Parameters of Animals after Immunization

The results of the hematological analysis are shown in Tables S2–S4. In the group immunized with FluCoVac-96, the level of hemoglobin and the number of basophils were decreased on D3 compared to D0, and then the levels returned to the original values on D28. In this group, significant differences were also observed in the percentage of monocytes on day 28, while the indicators remained within the reference values. In the group of monkeys that received the control H3N2 LAIV, there were significant differences in red blood cell counts, hemoglobin and hematocrit on day 28 compared to day 3. However, similar changes in these parameters were also found in mock-immunized monkeys, suggesting that these changes were not a consequence of drug administration but were due to changes in individual animals during their care. There were also no statistically significant differences between any of the three groups.

The results of blood biochemical parameters assessment are presented in Tables S5–S7. The elevated levels of alkaline phosphatase are normal for monkeys from the Research Institute of Medical Primatology and are of a nutritional nature. High levels of lactate dehydrogenase (LDH) were also noted, and in the mock-immunized group there was a trend toward a significant increase in this indicator compared to the initial values. A change in the level of LDH in the blood can indicate unclassified pathologies, as well as a number of natural, non-pathological processes (for example, active muscle function), and it is not possible to determine what exactly is disturbed. Otherwise, no changes in biochemical parameters from baseline values were observed. Statistical analysis of the biochemical parameters of the monkeys by group did not reveal any differences between the samples studied, and significant differences in the vaccine groups from baseline values for a number of indicators were also found in the placebo control group.

The results of coagulometry in experimental animals did not show any changes in APTT, fibrinogen and prothrombin time compared to baseline values (Table S8). In the FluCoVac-96-immunized group, there was a significant difference in fibrinogen levels on day 28 compared to baseline. In the H3N2 LAIV group, there were significant differences in prothrombin time values on day 28 compared to background values. Similar significant differences in fibrinogen levels were observed in the control group on day 28 of the experiment. No significant differences were found between the hemostasis parameters of the monkeys in all three groups.

In general, the study did not reveal a toxic effect of intranasally administered vaccines on the physiological parameters of monkeys through hematological analysis nor coagulation hemostasis. Immunization did not cause changes in the dynamics of the average weight and thermometry of the experimental animals. Thus, a preclinical study of the safety of the bivalent vaccine for the prevention of influenza and coronavirus infections in an experiment on rhesus monkeys showed that two-dose intranasal administration of the vaccines had no toxic effect on the experimental animals in any of the groups.

#### 3.2.2. Replication of Vaccine Viruses in the Upper Respiratory Tract of Monkeys

Virus replication in the upper respiratory tract was assessed by titration of nasal/throat swab material in eggs and real-time RT-PCR of the same samples. We observed very low levels of virus replication in the upper respiratory tract of rhesus macaques: viable virus was isolated from only one animal in the FluCoVac-96 group (No. 45884) on day 3 after immunization from a nasal swab, and virus genetic material was also detected in swabs from one animal of the H3N2 LAIV group (No. 45841) on day 1 in nasal swab and day 2 in throat swab material. In the remaining animals, active virus replication was not detected by standard virological methods, which is consistent with previously published data on the replicative activity of the vaccine strain of similar live influenza vaccine FluMist subtype H1N1 in macaques [22]. The endpoint RT-PCR revealed the presence of residual virus RNA material in the nasal swabs of animals from both vaccinated groups at day 1. Virus sequences from positive swabs were verified by Sanger sequencing. No virus replication was detected after the booster vaccine dose.

### 3.3. Immunogenicity of the FluCoVac-96 Strain in Rhesus Monkeys

Immunogenicity of the study vaccines was evaluated by measuring serum antibody responses to influenza virus on days 28 and 49 (4 weeks after each dose), and assessment of T-cell responses to influenza virus and SARS-CoV-2 antigens on day 35 (7 days after the second immunization).

#### 3.3.1. Serum Antibodies to Influenza Virus

**Figure 5:**
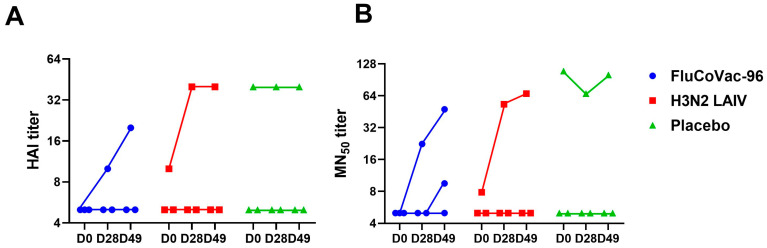
Serum antibody immune responses in rhesus macaques immunized with study vaccines or placebo. (**A**) Hemagglutination inhibiting antibodies to whole H3N2 LAIV virus. (**B**) Neutralizing antibodies against wild-type H3N2 virus. Antibody levels were measured at baseline (D0), after first immunization (D28) and after two doses (D49).

**Figure 6:**
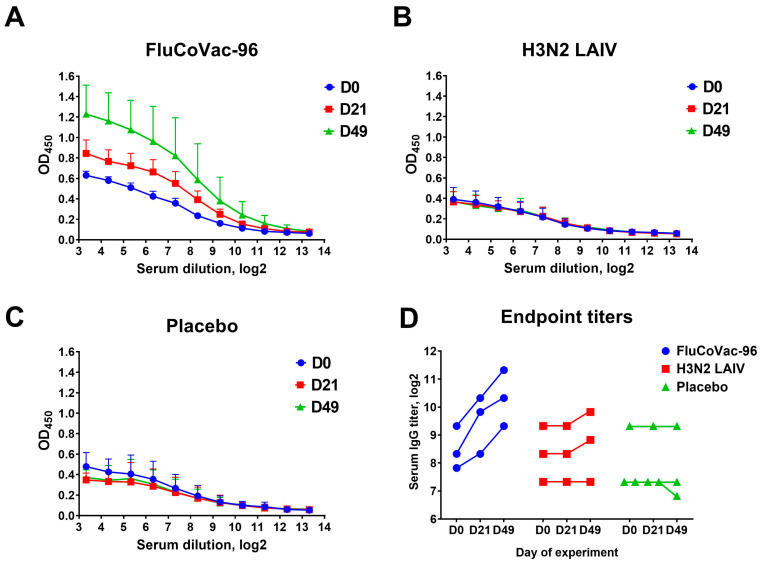
Serum IgG antibody levels in rhesus macaques immunized with study vaccines or placebo. (**A**) OD_450_ values for FluCoVac-96 vaccine group. (**B**) OD_450_ values for H3N2 LAIV group. (**C**) OD_450_ values for placebo group. (**D**) Endpoint serum IgG titers for each group at three time points. ELISA was performed with whole sucrose gradient-purified H3N2 LAIV virus. Antibody levels were measured at baseline (D0), after first immunization (D28) and after two doses (D49).

Influenza-specific serum antibody responses were measured by HAI assay, microneutralization assay and ELISA. Several monkeys in all study groups showed some levels of pre-existing influenza antibodies, which may be due to previous influenza exposure, as the monkeys were 3–7 years old (Figure 5 and Figure 6). Only one of three animals in each vaccine group had significant increases in HAI and MN_50_ titers (Figure 5), which is likely due to the inability of the H3N2 LAIV vaccine strain to actively replicate in the upper respiratory tract of rhesus macaques. No responses were seen in the placebo group. Before the start of the experiment, the screening of animals’ sera for the presence of antibodies to influenza was performed. One of the animals in the placebo group had to be urgently replaced by another one on the day of the start of the experiment, and later we detected that this monkey was seropositive for the flu virus, without any increase in titers throughout the experiment (Figure 5).

**Figure 7:**
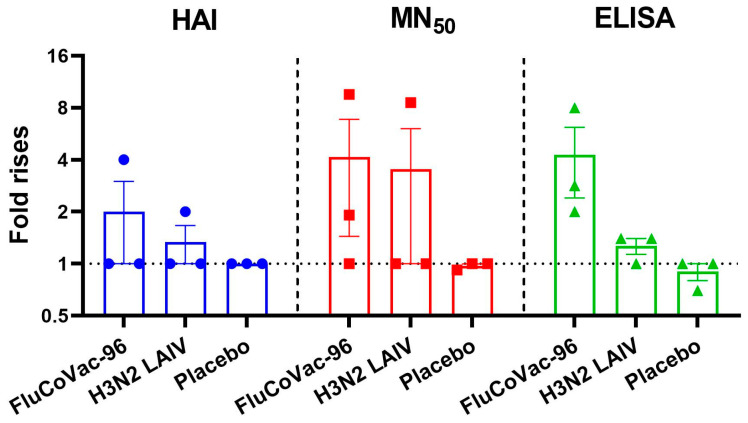
Fold rises of antibodies to influenza virus at D49 of the experiment in rhesus macaques immunized with study vaccines or placebo. HAI—hemagglutination inhibition; MN_50_—microneutralization test; ELISA—serum IgG antibodies.

Nevertheless, all three animals immunized with FluCoVac-96 had detectable increases in the levels of serum IgG antibodies in ELISA, whereas in the H3N2 LAIV-immunized group there was no increase in antibody responses (Figure 6). Overall, antibodies to influenza virus were low in both LAIV groups; however, it was noted that modification of the H3N2 LAIV virus by insertion of the SARS-CoV-2 T-cell cassette into its genome enhanced the antibody response to influenza virus as evidenced by ELISA results (Figure 7). The increase in immunogenicity of the modified vaccine strain compared to the classical variant corresponds to our earlier findings on the enhancement of immunogenic properties of LAIV strains when immunogenic T-cell epitopes of other respiratory viruses are integrated into their genome [21,59,60,61].

#### 3.3.2. Cell-Mediated Immune Responses to SARS-CoV-2 and Influenza Virus

T-cell responses to influenza virus and SARS-CoV-2 antigens were assessed by ELISPOT and ICS assays in PBMC samples collected on day 35 of the experiment.

##### IFN-γ FluoroSpot Assay

**Figure 8:**
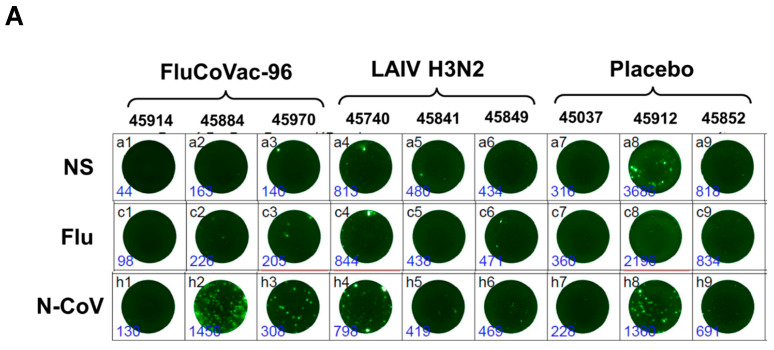

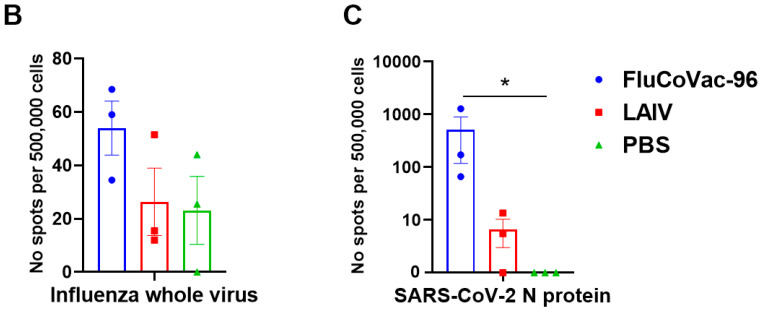
The levels of IFNγ-producing cells in PBMCs of studied monkeys after stimulation with influenza virus of recombinant SARS-CoV-2 N protein at D0 and D35 (7 days after the boost immunization) of the experiment. (**A**) Representative wells, FluoroSpot (IFNγ-FITC). (**B**) Levels of IFNγ-producing cells after stimulation with influenza virus; (**C**) stimulation with SARS-CoV-2 N. (*) *p* < 0.05 (Kruskal-Wallis test).

A variable baseline level of IFNγ production was observed in the PBMC of monkeys, which was probably associated with the individual interferon status of each animal. In particular, a very high background IFNγ was observed in macaque No. 45,912 of the mock-immunized group (Figure 8), this could indicate any ongoing infection or another physiological process. Stimulation of PBMCs with live influenza virus showed that the levels of influenza-specific cytokine-producing cells were higher in the FluCoVac-96 group compared to the two control groups, but the differences were not statistically significant due to the small number of animals per group. Stimulation with recombinant SARS-CoV-2 N protein revealed significantly higher levels of IFNγ-producing cells in the FluCoVac-96 group but not in the LAIV or placebo groups (Figure 8C). This may indicate the development of a T-cell response to SARS-CoV-2 after immunization with a recombinant LAIV strain encoding a coronavirus polyepitope cassette. It should be noted that the anti-TNFα antibodies used in the FluoroSpot Plus: Human IFN-γ/TNF-α kit did not cross-react with monkey TNFα, unlike the anti-IFNγ antibodies. Therefore, it was not possible to detect the levels of TNFα-producing cells in this experiment.

##### Intracellular Cytokine Staining with Flow Cytometry

Peripheral blood memory T-cell populations were assessed before immunization and 7 days after the boost dose with intracellular cytokine staining followed by flow cytometry. The gating strategy is shown in Figure S1. The distribution pattern of the lymphocyte subpopulations did not change after immunization and was stable for each animal (Figures S2 and S3). Monkey №45852 was shown to be immune to influenza virus at D0 and had an abnormal distribution of CD8 T-cell memory subsets and was therefore excluded from the flow cytometry experiment data processing (Figure S2). We analyzed cytokine-producing CD4+ and CD8+ T cells with CD45RA- phenotype (memory T cells), with CD197+/− subpopulations. In many studies, the molecules used for immunophenotyping of memory subsets are the same as for human cells: CD45RA and CCR7 (CD197) [51,62,63]. The careful analysis of the papers and of our own data revealed that these memory subsets are less characteristically separated in monkeys using these markers than they are in humans (Figure S4), so we analyzed not only effector memory subset CD45RA-CD197- but also the entire pool of memory CD45RA- T cells. To exclude possible technical error in memory subset calculation, before the experiment we compared the staining of two monkeys’ PBMCs with two different antibody panels (Figure S4). The subsets percentage was the same as in the subsequent experiment.

**Figure 9:**
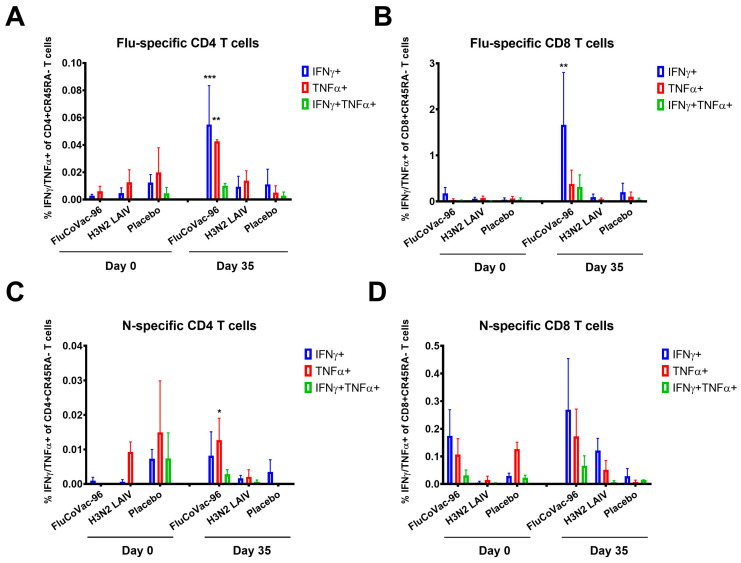
The levels of IFNγ/TNFα-producing T cells in PBMCs of rhesus macaques before the immunization (day 0) and 7 days after the boost dose (day 35). (**A**) The levels of CD4+ memory T cells specific to influenza virus. (**B**) The levels of CD8+ memory T cells specific to influenza virus. (**C**) The levels of CD4+ memory T cells specific to SARS-CoV-2 N protein. (**D**) The levels of CD8+ memory T cells specific to SARS-CoV-2 N protein. The statistically significant differences between levels at day 0 and day 35 are shown (two-way ANOVA, uncorrected Fisher’s LSD test for multiple comparisons * *p* < 0.05; ** *p* < 0.01; *** *p* < 0.001).

**Figure 10:**
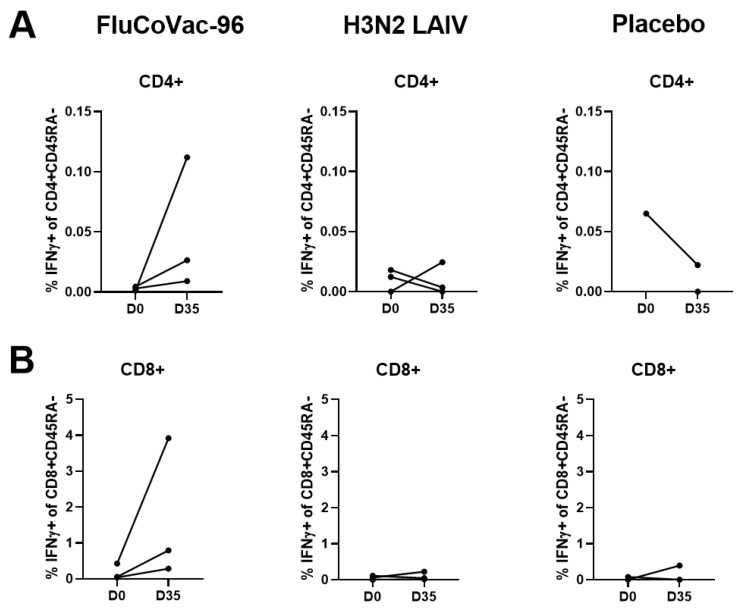
The levels of IFNγ-producing T cells in PBMCs of rhesus macaques after H3N2 influenza virus stimulation. (**A**) CD4+ CD45RA- T-cells subset. (**B**) CD8+ CD45RA- T-cells subset.

**Figure 11:**
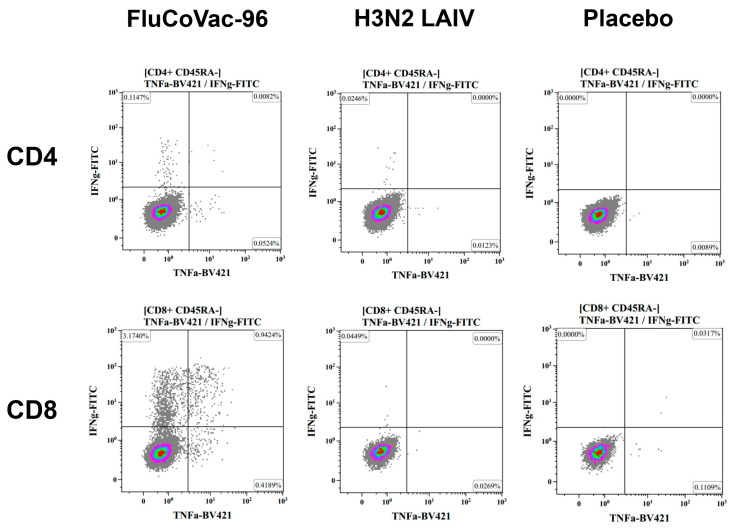
Representative gates of CD4+ CR45RA- (top panel) and CD8+ CR45RA- (bottom panel) IFNγ/TNFα-producing T cells after stimulation of the PBMCs with live purified influenza H3N2 virus.

**Figure 12:**
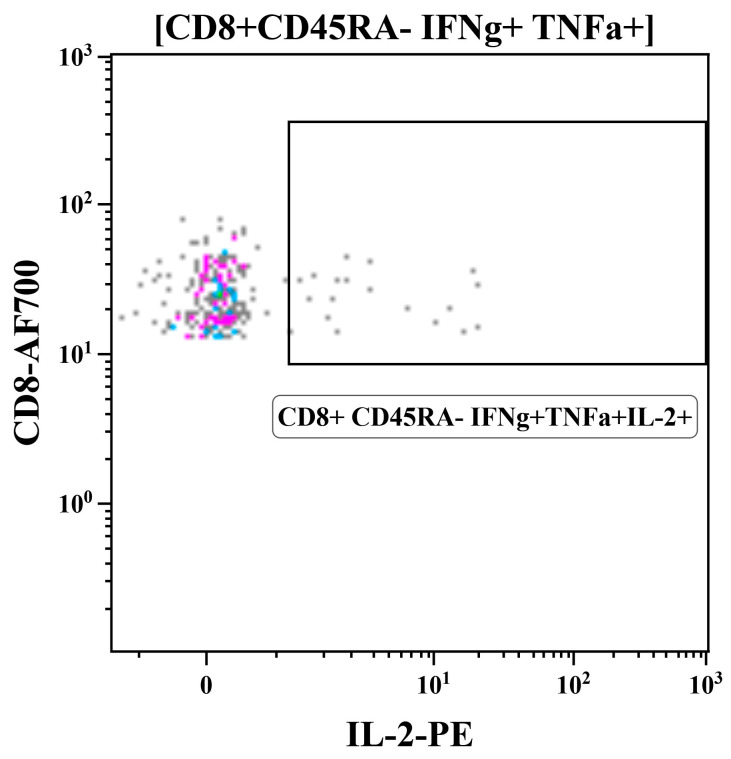
The polyfunctional memory T cells (CD8+ CD45RA- IFNγ+ TNFα+ IL-2+) in PBMCs of macaque #45884 immunized with FluCoVac-96 after influenza stimulation (day 35 of the experiment). The percentage of IFNγ+ TNFα+ IL-2+ cells of CD8+ CD45RA- population is 0.0686%.

The summarized data on the levels of cytokine-producing memory T cells prior to immunization and 7 days after the boost dose are shown in Figure 9. Both CD4+ and CD8+ influenza-specific CD45RA- cells were detected at higher levels in the FluCoVac-96-immunized group than in the H3N2 LAIV immunized group (Figure 9A,B and Figure 10). This is consistent with our previous findings: the incorporation of foreign T-cell epitopes into the genome of influenza vaccine strains leads to an enhancement of the immunogenicity of the strain to influenza virus [61]. Representative gates are shown in Figure 11, and the CD45RA- cells, producing IFNγ, TNFα or both cytokines are gated on the plots. The most pronounced T-cell response was seen in monkey №45884, which had elevated antibody titers to influenza virus and in which virus shedding was observed. In the PBMCs of animal №45884, we also detected the presence of polyfunctional memory T cells (IFNγ + TNFα + IL-2+) (Figure 12). The presence of cells with this phenotype is a marker for the formation of a long-lived memory T-cell population that can respond rapidly to pathogen reinvasion [64,65]. Cells with this phenotype are extremely rare in peripheral blood samples [64].

**Figure 13:**
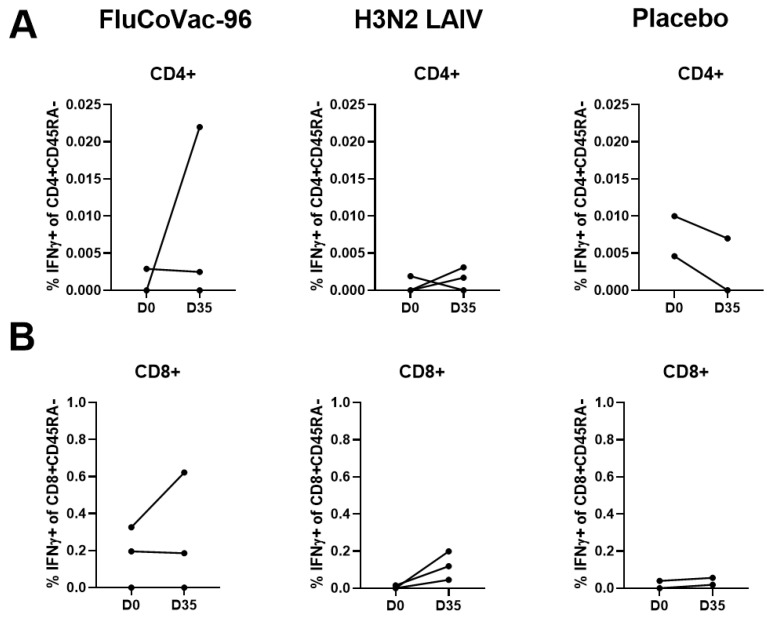
The levels of IFNγ-producing T cells in PBMC of rhesus macaques after recombinant SARS-CoV-2 N protein stimulation. (**A**) CD4+ CD45RA- T-cells subset. (**B**) CD8+ CD45RA- T-cells subset.

Stimulation of PBMCs with recombinant SARS-CoV-2 N protein showed significantly lower response in immunized groups compared to influenza virus (Figure 9C,D and Figure 13). A significant increase in TNFα-producing CD4+ memory T cells specific for SARS-CoV-2 N was also observed in the group immunized with FluCoVac-96 (Figure 9C). On day 35 of the experiment, levels of N-specific T cells were higher in the FluCoVac-96-immunized animals compared to the mock immunized group (Figure 9 and Figure 13). These data suggest that the bivalent vaccine stimulates not only influenza-specific T-cell immunity but also immunity to SARS-CoV-2 epitopes. The most pronounced increase in N-specific CD4 and CD8 CD45RA memory T cells was also detected in monkey №45884, which was immunized with FluCoVac-96 and had a good response to the influenza virus. This suggests that active replication of the recombinant vaccine virus in the monkey’s URT can stimulate the induction of systemic T-cell immunity to both the influenza virus and to epitopes of the N protein partially contained in the polyepitope cassette embedded in the NA gene of the influenza vaccine virus.

### 3.4. Protection against Challenge with SARS-CoV-2 Virus

To assess the impact of immunization with the bivalent vaccine on subsequent SARS-CoV-2 infection, the animals were experimentally challenged with 5 lg PFU of SARS-CoV-2 Delta virus by the intranasal route. The Delta strain has rapid disease spread and causes pathogenic changes in rhesus monkeys, and the dose selection was based on other published studies [66,67,68]. The virus was administered dropwise using a standard pipette tip. Animals were monitored for 6 days following challenge (behavior, temperature, body weight and nasal and rectal swabs to detect virus shedding). On day 6, the animals were humanely euthanized, and their organs were removed for examination.

**Figure 14:**
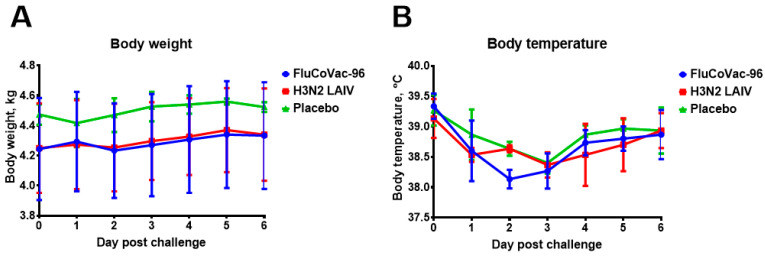
The body weight and body temperature monitoring in groups of rhesus macaques after SARS-CoV-2 intranasal challenge. (**A**) Average body weight dynamics in groups. (**B**) Body temperature dynamics.

No effect of SARS-CoV-2 challenge on body weight and body temperature was observed in any of the groups (Figure 14). None of the rectal temperatures of any of the animals exceeded what is normal for monkeys (39.5 °C), and any differences between groups were within the individual variations and were not statistically significant.

**Figure 15:**
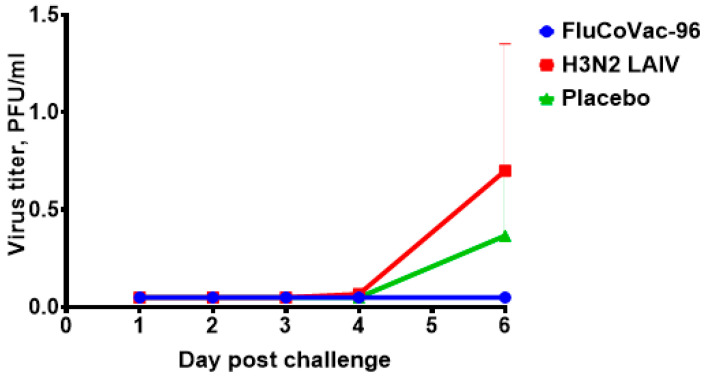
Titers of live SARS-CoV-2 virus in the respiratory organs of the rhesus macaques after challenge infection.

Live SARS-CoV-2 virus was isolated only in the H3N2 LAIV-immunized group and the mock-immunized group (the differences in titers were not statistically significant) (Figure 15). On day 6 post-challenge, the animals were humanely euthanized, and lung tissue samples were examined for virus presence by real-time PCR assay and titration in Vero cells. No infectious virus was observed in the lungs of the animals on day 6 post-challenge. The absence of intense viral replication in the sham-immunized group indicated that it would be inappropriate to evaluate the protective potential of the vaccine candidates studied by virological endpoint. However, the immunization with the modified vaccine strain did not result in an enhancement of the coronavirus disease (Figure 14 and Figure 15).

#### Histopathology Studies

Histological examination of lung tissue samples revealed that the vascular system was unevenly full of blood and the airways were passable. There were areas of emphysematous expansion of the alveoli, alternating with foci of bronchogenic and perivasal inflammatory consolidation. The severity of changes in the lung tissue was lower in the samples of animals immunized with FluCoVac-96 and H3N2 LAIV, compared to mock-immunized animals (Figure S5). In immunized animals, there were foci of interstitial pneumonia in samples, while in the placebo group the interstitial pneumonia was confluent, and bronchiolitis was detected in mock-immunized animals. In samples of the spleen, kidneys, heart and trachea of inspected animals, there were no deviations from species rate. The liver samples of animals from the groups immunized with H3N2 LAIV or a placebo contained single small mixed cell granulomas consisting of leukocytes, neutrophils and single histiocytes, although these findings had no clinical significance. The representative micrographs of the organs of the animals of all three groups are shown in Figure S5.

## 4. Discussion

Despite the end of COVID-19 pandemic, disease cases continue to be detected worldwide, so the development of vaccines providing complex immunity against such dangerous respiratory infections as influenza and COVID-19 is still relevant. Numerous studies have reported the correlation between the robust T-cell responses and a milder course of COVID-19 [1,2]. Thus, the establishment of a pool of lung tissue resident memory T cells specific for SARS-CoV-2 epitopes might be considered as a tool to prevent severe COVID-19. Previously, we proposed a vectored vaccine candidate based on a licensed strain of live attenuated influenza vaccine bearing the insertion encoding SARS-CoV-2 epitopes for targeted stimulation of T-cell immunity [21]. One of the tested prototypes appeared to be effective in mouse and hamster models. In the current study, we used the same SARS-CoV-2-based immunogenic cassette to insert into the NA gene of the licensed LAIV strain of H3N2 subtype. The H3N2 influenza strain is the obligatory component of the seasonal influenza vaccines, having a high variability rate, and therefore should be updated almost every season. The strain retained mandatory in vitro characteristics of live influenza vaccine, i.e., the ability to propagate in eggs and cell cultures, a cold-adapted phenotype and a reduced ability to replicate at high temperatures. The genetic stability of the strain was demonstrated after ten egg passages. This allowed the safety of the strain to be assessed in non-human primates. Of note, the FluCoVac-96 strain was safe and immunogenic for Syrian hamsters when administered alone or in combination with H1N1 LAIV carrying the same SARS-CoV-2 cassette, as well as with unmodified LAIV type B. Such a trivalent LAIV/CoV-2 composition is being developed as a seasonal vaccine capable of protecting against all circulating human influenza viruses (manuscript in preparation).

The rhesus macaque model was selected for the bivalent vaccine study [35,69] as appropriate for both influenza and SARS-CoV-2 viruses. Several influenza subtypes have been previously studied in this model: H1N1 [22,23,24], including live attenuated vaccine [22], H3N2 [25,26,27,28] and H5N1 [29]; studies prior to 2014 are reviewed in [30]. Rhesus macaques have also been shown to be susceptible to SARS-CoV-2 [31,32,33,34,35], including the Delta subvariant used in our study [66,67,68,70]. Rhesus monkeys have been used in studies of influenza vaccines and in studies of anti-COVID-19 drugs and vaccines [36,37,38,71,72,73,74], including the mucosal one [72].

In our study, the observed shedding of vaccine virus was extremely low for both modified and unmodified LAIV. These results are consistent with data previously obtained for a cold-adapted H1N1 subtype influenza vaccine [22], demonstrating that despite replicating the virus was not detected in nasal swabs, and viral replication in nasal turbinates was observed. The current study represents the first published data on cold-adapted H3N2 influenza virus shedding in a rhesus macaque model. Previously published data describe propagation of H3N2 influenza strains in a rhesus model, in particular A/Aichi/2/1968 model virus [26,28] and seasonal A/Sydney/5/97 strain [27]. In all cases, virus titers in nasal washes were low, not exceeding 10^4^/mL on day 3 for A/Aichi/2/1968 [26] and closing to the detection limit (10^1.5^/mL) for the strain A/Sydney/5/97 [27]. In our study, the most pronounced viral replication in nasal swab was detected in animal #45884, which obviously correlated with robust humoral and T-cell responses.

Here, serum antibodies were assessed only to influenza virus, because the insertion consisted of SARS-CoV-2 epitopes undergoing intracellular processing for T-cell response stimulation. An enhanced immunogenicity of the modified strain was observed compared to the LAIV, which is in good agreement with our previous results, demonstrating the enhancement of LAIV immunogenicity after incorporation into the viral genome of T-cell-targeted cassettes encoding the antigens of other respiratory pathogens [21,59,60,61].

The studied SARS-CoV-2 cassette contains the relatively prolonged fragments of the N protein and conservative regions of the S antigen, so despite it being designed for human T-cell stimulation, the construction may also be used to trigger animal T-cell responses. The T-cell subpopulations of rhesus monkeys have been revealed in several studies [51,52,53,63]. We evaluated the T-cell responses 7 days after the booster dose by analyzing PBMCs using ELISPOT and ICS followed by flow cytometry. The N-specific cytokine-producing T cells, especially CD8+, were detected at higher levels in the FluCoVac-96-immunized group compared to the comparator groups. At the same time, no cytokine responses were detected when attempting peptide pool stimulation. The intensity of the T-cell responses in the group immunized with unmodified LAIV appeared to be unexpectedly low. This fact is in line with the previously demonstrated phenomenon that polyfunctional memory T cells can rarely be detected in peripheral blood [64]. Hypothetically, T-cell immune responses in mice may be more pronounced due to increased immunodominance in inbred animals. Analysis of the cell phenotype in central and peripheral lymphoid organs, which is usually performed in mouse models, often demonstrates more representative immune response pattern than studying the peripheral blood lymphocytes [22,75]. In our study, correlation between the intensities of T-cell immune responses and vaccine virus replication was detected. However, the protection against a challenge virus could be provided even if the vaccine replication was not detected [22], and a number of non-replicating influenza-vectored vaccines with immunogenic potential were listed [16,17,19]. The impact of the T-cell immunity mode in anti-SARS-CoV-2 protection has been previously demonstrated by McMahan et al. in a rhesus monkey model [3] and by Kingstad-Bakke et al. in a mouse model [76]. Thus, we found that both vectored vaccines stimulated the formation of memory T cells, and LAIV modification results in enhanced T-cell response to influenza virus. More detailed experiments are needed to analyze the mechanisms of the vaccine-induced immune responses in barrier tissues.

The clinical manifestations of SARS-CoV-2 infection in rhesus macaques described in the literature are variable, dependent on age [34,77], virus dose [67] and the virus inoculation route [33]. The most evident consequence of the infection in rhesus macaques is interstitial pneumonia [78]. Weight loss and fever are usually rare symptoms which may be detected late in the course of the infection [32]. Nasal SARS-CoV-2 shedding in rhesus monkeys is demonstrated to be dose-dependent [67]. We selected the Delta SARS-CoV-2 strain because it was demonstrated to cause pathogenic changes in a rhesus macaque model [67], compared to currently circulating Omicron variants, which are low-pathogenic for macaques [79]. The cassette composition is combined of relatively conservative fragments, so we expected that changing the strain would not change the effect of the vaccine administration.

The absence of Vaccine-Associated Enhanced Respiratory Disease (VAERD) is an important widely recognized safety marker. For the SARS-CoV-2 virus, this effect was first observed in a mouse model of infection [39], and the mechanism of antibody involvement was studied in cell cultures [80,81]. Besides the antibodies, other immune components may be implicated in VAERD—for respiratory syncytial virus and measles, the involvement of complement and T cells has been described (reviewed in [40]). For SARS-CoV-2, the leading role of type 2 immunopathology in VAERD has been demonstrated in a hamster model [41] and ACE2-humanized mice immunized with adjuvanted spike-protein vaccine [42]. The role of immunopathology in humans has also been studied [82,83]. The rhesus monkeys were used to assess antibody-dependent enhancement (ADE) of SARS-CoV-2 infection during vaccine testing [84]. Here, we did not observe any enhancement of disease induced by challenge of monkeys with the Delta sublineage of SARS-CoV-2, which was evaluated either for clinical symptoms or for viral replication and histopathology changes in organs at the 6th day after viral inoculation. Although a low level of viral replication in the control group made it inappropriate to rate the protective effect on this indicator, the histopathology study demonstrated the protective effect of immunization: in the mock-immunized animals, the interstitial pneumonia was confluent in contrast to the monkeys from the immunized groups.

This study has several limitations. The number of animals per group was small, which imposes limitations on the statistical analysis and enhances the influence of random events. The animals were 3–4 years old, and the status of possible previous asymptomatic infections with influenza and SARS-CoV-2 viruses was assessed only by serum antibody level. Serum antibody levels are known to decline over time [85], as do the number of circulating memory cells [86,87]; at the same time, the level of local immune response in the respiratory tract can remain intense. It is known that infection with SARS-CoV-2 protects rhesus macaques from re-infection [88,89]. In this regard, it is particularly important to note that immunization did not induce VAERD, as this is a baseline indicator of vaccine safety and allows further studies to be conducted. These features of monkeys as large model animals (age, immunological diversity, possibility of pre-existing immunity that are difficult to detect during standard screening), on the one hand, complicate the interpretation of the data obtained but on the other hand make the results of the experiment close to the real situation in the human population. In the current study, we did not assess the local immune response after SARS-CoV-2 challenge due to the impossibility to perform such studies in a BSL-3 facility during this experiment.

## 5. Conclusions

Our data suggest that the modified FluCoVac vaccine was safe and well tolerated in the non-human primate model, and there was no effect of vaccine-associated enhancement of disease after challenge with SARS-CoV-2 at a non-lethal dose. Administration of the vaccine did not cause changes in the clinical condition of the animals nor in the clinical and biochemical blood parameters. Vaccine virus shedding was low, which excludes the possibility of the reassortment of the vaccine strain with circulating influenza viruses. The modified vaccine stimulated antibody responses to the influenza virus as well as the formation of a pool of memory T cells. Subsequent challenge of the macaques with SARS-CoV-2 Delta virus did not result in severe disease in the immunized animals. Thus, this vaccine can be further pursued in clinical development.

## Data Availability

The data presented in this study are available on request from the corresponding author.

## Supplementary Materials

The following supporting information can be downloaded at: https://www.mdpi.com/article/10.3390/vaccines12101099/s1, Table S1: Clinical examination parameters; Table S2: Hematological parameters of experimental animals of FluCoVac-96 group; Table S3: Hematological parameters of experimental animals of H3N2 LAIV group; Table S4: Hematological parameters of experimental animals of placebo group; Table S5: Blood biochemical parameters of animals of the FluCoVac-96 group; Table S6: Blood biochemical parameters of animals of the H3N2 LAIV group; Table S7: Blood biochemical parameters of animals of the placebo group; Table S8: Coagulation hemostasis indicators of rhesus macaques in dynamics; Table S9: Monitoring of body weight and rectal temperature of monkeys after infection with SARS-CoV-2 virus, Delta strain; Figure S1: Gating strategy for immunophenotyping of cytokine-producing T-cell subsets in rhesus monkey PBMCs; Figure S2: CD8+ memory T-cell subsets distribution in individual animals’ PBMCs; Figure S3: CD4+ memory T-cell subsets distribution in individual animals’ PBMCs; Figure S4: Memory T-cell subsets stained with different antibody panels; Figure S5: Representative micrographs of monkeys’ lung tissue fragments.
