# Supplementary material for: Safety and Immunogenicity Study of a Bivalent Vaccine for Combined Prophylaxis of COVID-19 and Influenza in Non-Human Primates
Source: Vaccines (Basel). 2024 Sep 26;12(10):1099. doi: 10.3390/vaccines12101099 (PMC11511058; doi:10.3390/vaccines12101099)
Supplement: Supplementary file 1 [file vaccines-12-01099-s001.zip › vaccines-3190033-supplementary.pdf]

SUPPLEMENTARY

Safety and immunogenicity study of a bivalent vaccine for combined prophylaxis of COVID-19 and influenza in non-human primates

Ekaterina Stepanova[1], Irina Isakova-Sivak[1], Victoria Matyushenko[1], Daria Mezhenskaya[1], Igor Kudryavtsev[1], Arina Kostromitina[1], Anna Chistiakova[1], Alexandra Rak[1], Ekaterina Bazhenova[1], Polina Prokopenko[1], Tatiana Kotomina[1], Svetlana Donina[1], Vlada Novitskaya[1], Konstantin Sivak[2], Dzhina Karal-Ogly[3], Larisa Rudenko[1]

Table S1 - Clinical examination parameters

Group of parameters	Indicator
**External appearance**	normal
	abnormal
**Skin and coat condition**	normal
	ruffled hair
	hair loss
	hair growth
	loss of gloss
	mottling
	abrasions
	skin fissures
	injuries
	skin dryness
**Eyes condition**	normal
	lacrimation
	eye suppuration
	eye inflammation
	eye swelling
**Nasal condition/breathing**	normal
	shallow breathing
	intermittent breathing
	dyspnoea
	nasal discharge
	haemorrhages
	oedema
**Stool condition**	normal
	stool discolouration
	presence of blood in the stool
	Gruel stool
	diarrhoea
	lack of stool
**Appetite**	normal
	reduced appetite
	lack of appetite
**Mucous membranes**	normal
	mucous membrane redness
	pallor of mucous membranes
	lividity of mucous membranes
	mucous membrane jaundice
**Body positioning**	normal
	posture loss
	forced lying down
	forward and backward motion
	compulsive tendency to lie on one side
	contracture

Group of parameters	Indicator
**Behavior and movement coordination**	normal
	excitement
	sluggishness
	aggression
	reduced activity
	hyperkinesis
	coordination disorder
	twitching
	tremor
	seizures
	retropulsion
	paresis
	immobility

Table S2- Haematological parameters of experimental animals of FluCoVac-96 group

ID	Haematological test results													
	Day of the experiment	Clinical blood test indicators								Leukocyte count, %				
		RBC,× 10^{12}/l (5,00-6,20)	Hgb, g/l (110-145)	Hct, % (26,0 – 45,0)	MCV, fl (52,0 – 97,0)	MCH, pg (18,0 – 33,0)	MCHC, g/l (238 – 442)	Plt, × 10^9/l (200 – 600)	WBC, × 10^9/l (5,5 – 13,0)	MO (1,0-5,0)	LY (25,0-55,0)	NE (35,0-65,0)	EO (1,0-5,0)	BA (0-2,0)
45914	0	5,41	128	40,2	74,3	23,7	318	402	11,6	4,6	40,2	54,2	0,2	0,8
	3	4,90	113	36,6	74,7	23,1	309	265	10,1	3,1	41,4	55,0	0,2	0,3
	28	6,40	146	48,3	75,5	22,8	302	378	10,8	5,3	47,4	46,8	0,2	0,3
	31	5,17	121	38,8	75,0	23,4	312	273	16,3	6,1	36,6	56,9	0,0	0,4
45884	0	6,84	144	47,1	68,9	21,1	306	233	9,8	3,8	34,9	60,4	0,2	0,7
	3	5,73	117	39,9	69,6	20,4	293	325	13,6	2,6	27,8	68,8	0,3	0,5
	28	6,14	127	42,9	69,9	20,7	296	286	8,1	5,1	52,7	40,6	0,2	1,4
	31	5,21	114	36,1	69,3	21,9	316	305	6,2	4,0	58,7	36,0	0,2	1,1
45970	0	5,82	137	42,8	73,5	23,5	320	388	11,3	4,4	32,9	60,5	1,6	0,6
	3	5,66	127	41,8	73,9	22,4	304	340	6,0	6,2	45,6	46,6	1,2	0,4
	28	5,86	136	43,3	73,9	23,2	314	398	8,6	5,0	33,3	60,1	1,2	0,4
	31	5,05	116	37,4	74,1	23,0	310	371	7,7	5,7	35,9	56,4	1,4	0,6

Table S3- Haematological parameters of experimental animals of H3N2 LAIV group

ID	Haematological test results													
		Clinical blood test indicators								Leukocyte count, %				
	Day of the experiment	RBC,× 10^{12}/l (5,00-6,20)	Hgb, g/l (110-145)	Hct, % (26,0 – 45,0)	MCV, fl (52,0 – 97,0)	MCH, pg (18,0 – 33,0)	MCHC, g/l (238 – 442)	Plt, × 10^9/l (200 – 600)	WBC, × 10^9/l (5,5 – 13,0)	MO (1,0-5,0)	LY (25,0-55,0)	NE (35,0-65,0)	EO (1,0-5,0)	BA (0-2,0)
45740	0	6,69	151	48,5	72,5	22,6	311	248	13,5	2,4	26,6	69,7	0,7	0,6
	3	5,45	127	40,2	73,8	23,3	316	248	8,2	1,6	36,1	61,1	0,7	0,5
	28	6,59	152	49,5	75,1	23,1	307	319	9,3	1,7	42,1	55,6	0,2	0,4
	31	5,77	131	43,2	74,9	22,7	303	310	8,4	3,6	49,3	46,0	0,4	0,7
45841	0	5,72	123	38,7	67,7	21,5	318	416	10,9	3,9	41,3	50,0	3,8	1,0
	3	5,50	115	37,4	68,0	20,9	307	517	12,0	2,9	44,1	50,3	2,3	0,4
	28	7,08	148	48,7	68,8	20,9	304	395	15,9	5,3	45,2	46,3	2,9	0,3
	31	6,03	128	41,7	69,2	21,2	307	359	11,1	6,8	57,3	32,9	2,7	0,3
45849	0	6,60	147	47,4	71,8	22,3	310	290	9,1	7,1	41,7	48,5	1,5	1,2
	3	5,65	123	40,3	71,3	21,8	305	259	11,1	4,7	40,9	52,6	1,1	0,7
	28	6,47	143	46,1	71,3	22,1	310	302	6,7	5,6	51,1	40,9	1,2	1,2
	31	6,12	134	43,8	71,6	21,9	306	224	7,4	6,3	42,7	49,0	0,9	1,1

Table S4- Haematological parameters of experimental animals of Placebo group

ID	Haematological test results													
		Clinical blood test indicators								Leukocyte count, %				
	Day of the experiment	RBC,× 10^{12}/l (5,00-6,20)	Hgb, g/l (110-145)	Hct, % (26,0 – 45,0)	MCV, fl (52,0 – 97,0)	MCH, pg (18,0 – 33,0)	MCHC, g/l (238 – 442)	Plt, × 10^9/l (200 – 600)	WBC, × 10^9/l (5,5 – 13,0)	MO (1,0-5,0)	LY (25,0-55,0)	NE (35,0-65,0)	EO (1,0-5,0)	BA (0-2,0)
45037	0	6,62	141	47,8	72,2	21,3	295	362	10,0	4,5	33,0	61,2	0,5	0,8
	3	5,71	124	40,9	71,6	21,7	303	380	6,9	3,3	44,3	49,5	2,3	0,6
	28	6,72	150	48,4	72,0	22,3	310	289	5,4	4,2	56,6	36,7	1,3	1,2
	31	6,38	138	46,1	72,3	21,6	299	270	10,9	4,9	50,2	39,7	4,4	0,8
45912	0	6,07	134	42,6	70,2	22,1	315	226	12,8	5,2	30,4	63,3	0,5	0,6
	3	5,52	121	39,2	71,0	21,9	309	201	9,2	2,3	39,7	57,0	0,4	0,6
	28	6,36	138	44,4	69,8	21,7	311	115	14,2	2,8	27,4	69,1	0,3	0,4
	31	5,69	124	40,6	71,4	21,8	305	158	16,2	5,0	24,5	69,5	0,3	0,7
45852	0	6,16	133	43,1	70,0	21,6	309	379	12,2	5,0	51,3	42,8	0,4	0,5
	3	5,49	119	38,9	70,9	21,7	306	273	12,8	3,4	50,5	44,6	1,2	0,3
	28	6,57	141	46,4	70,6	21,5	304	358	9,5	2,3	72,7	24,2	0,2	0,6
	31	5,35	114	37,9	70,8	21,3	301	355	10,0	3,3	60,1	35,5	0,6	0,5

Table S5- Blood biochemical parameters of animals of the FluCoVac-96 group

ID	Day of the experiment	GLU (3,9-6,1) mmol/l	ALP (98-300) U/l	ALT (0-40) U/l	AST (0-40) U/l	ALT / AST (1,0-1,2)	LDH (240-480) U/l	TB (2-20) mmol /l	UREA (2,5-7,5) mmol/l	CRE (53-115) mmol /l	CHOL (3,6-5,5) mmol/l	TG (0,3-1,85) mmol /l	TP (60-85) g/l	K (3,0-6,0) mmol /l	Na (100-150) mmol /l
45914	0	5,7	642	116	39	3,0	1134	13,8	5,0	143	3,06	0,54	82	5,5	105
	3	8,9	678	96	41	2,3	1033	11,3	5,2	174	3,12	0,43	79	5,9	110
	28	3,8	595	39	52	0,8	1105	15,6	5,8	199	4,49	2,62	84	5,7	103
	31	10,9	506	155	84	1,8	567	8,4	5,5	177	3,21	0,65	83	7,8	104
54884	0	6,4	840	34	32	1,1	621	16,5	5,6	107	3,95	0,86	75	4,2	106
	3	5,0	923	61	36	1,7	617	7,0	6,0	116	4,15	0,34	76	42	94
	28	6,1	483	28	26	1,1	310	7,3	6,0	113	5,86	0,38	71	3,7	107
	31	7,4	483	27	37	0,7	729	9,4	5,5	119	4,96	0,41	72	3,7	101
45970	0	4,9	965	36	34	1,1	678	15,7	5,6	131	3,34	0,70	77	4,6	109
	3	5,8	946	76	31	2,5	758	11,3	5,6	137	3,68	0,64	78	5,4	106
	28	5,5	409	30	21	1,4	336	5,7	6,0	141	4,37	1,00	71	5,3	107
	31	7,8	736	38	37	1,0	862	5,8	5,2	159	3,62	0,73	77	4,4	109

Table S6- Blood biochemical parameters of animals of the H3N2 LAIV group

ID	Day of the experiment	GLU (3,9-6,1) mmol/l	ALP (98-300) U/l	ALT (0-40) U/l	AST (0-40) U/l	ALT / AST (1,0-1,2)	LDH (240-480) U/l	TB (2-20) mmol /l	UREA (2,5-7,5) mmol/l	CRE (53-115) mmol/l	CHOL (3,6-5,5) mmol/l	TG (0,3-1,85) mmol/l	TP (60-85) g/l	K (3,0-6,0) mmol/l	Na (100-150) mmol /l
45740	0	4,6	606	37	23	1,6	641	12,5	6,1	128	3,33	0,41	71	4,8	103
	3	3,5	621	62	45	1,4	948	7,3	5,8	122	3,51	0,17	67	4,1	109
	28	5,9	453	40	25	1,6	439	7,7	6,5	144	4,87	0,34	70	3,1	92
	31	6,5	398	47	36	1,3	1123	5,8	5,4	133	3,79	0,51	69	4,7	104
45841	0	5,0	1030	39	22	1,8	843	16,1	6,0	118	4,08	0,58	71	5,2	107
	3	6,8	1389	43	36	1,2	973	9,8	6,0	127	3,81	0,36	75	4,6	108
	28	5,2	485	32	27	1,2	538	8,2	6,5	119	3,33	0,25	80	4,9	107
	31	8,6	733	32	38	0,8	991	9,8	5,7	129	5,94	0,31	76	4,2	111
45849	0	3,8	1126	30	17	1,8	551	6,7	5,9	120	2,63	0,32	66	4,5	110
	3	4,9	1066	30	21	1,4	649	7,3	6,3	130	1,64	0,15	61	3,8	103
	28	4,9	911	28	16	1,8	283	5,9	6,6	122	5,70	0,49	67	2,3	107
	31	5,5	845	25	24	1,0	487	5,2	5,4	132	4,88	0,57	74	4,0	108

Table S7- Blood biochemical parameters of animals of the Placebo group

ID	Day of the experiment	GLU (3,9-6,1) mmol/l	ALP (98-300) U/l	ALT (0-40) U/l	AST (0-40) U/l	ALT / AST (1,0-1,2)	LDH (240-480) U/l	TB (2-20) mmol /l	UREA (2,5-7,5) mmol/l	CRE (53-115) mmol/l	CHOL (3,6-5,5) mmol/l	TG (0,3-1,85) mmol/l	TP (60-85) g/l	K (3,0-6,0) mmol/l	Na (100-150) mmol/l
45037	0	3,2	1611	47	35	1,3	599	8,3	6,3	129	2,24	0,46	76	4,6	109
	3	3,5	1761	163	134	1,2	1154	9,7	6,2	138	2,71	0,53	73	5,1	107
	28	4,3	1065	35	30	1,2	456	6,7	6,4	139	6,06	0,64	78	3,4	105
	31	4,7	1104	52	39	1,3	810	4,5	5,7	139	4,80	0,77	77	5,5	105
45912	0	4,9	822	53	32	1,7	687	11,5	6,4	130	1,87	0,50	79	4,8	108
	3	4,7	851	76	83	0,9	1175	11,5	6,8	122	2,03	0,42	81	5,1	109
	28	5,0	468	71	40	1,8	503	10,2	6,8	137	6,80	0,46	76	3,8	109
	31	6,1	460	62	77	0,8	1167	8,8	5,7	135	5,65	0,31	74	5,4	110
45852	0	5,9	1060	35	23	1,5	697	9,1	6,5	137	3,65	0,47	76	5,3	110
	3	6,4	1180	49	50	1,0	1061	13,4	6,7	166	2,50	0,49	75	4,0	102
	28	8,6	675	30	24	1,3	469	9,0	6,2	163	5,85	0,59	71	4,3	108
	31	9,1	711	43	48	0,9	1183	9,0	5,6	147	5,38	1,06	73	4,4	110

Table S8 – Coagulation haemostasis indicators of rhesus macaques in dynamics

Group	ID	Day of the experiment	APTT, sec Ref. : 21-38 sec.	Fib, g/l (fib) Ref. : 2.00-4.00	Protrombin time, sec (pt) Ref. : 10-15 sec
FluCoVac-96	45914	0	22,0	2,37	13,6
		3	21,5	2,01	12,4
		28	21,4	2,03	12,3
		31	21,6	4,18	12,6
	45884	0	18,6	2,23	12,4
		3	18,4	2,67	11,1
		28	18,9	2,15	10,8
		31	19,5	3,48	11,3
	45970	0	20,9	2,48	14,0
		3	19,3	2,31	12,4
		28	19,1	2,00	11,6
		31	20,7	3,35	12,5
LAIV H3N2	45740	0	23,4	1,99	14,5
		3	21,4	2,73	12,9
		28	22,5	2,03	11,9
		31	21,6	2,98	11,6
	45841	0	22,5	2,97	12,8
		3	23,3	3,47	11,4
		28	23,5	2,56	11,8
		31	24,1	3,38	12,5
	45849	0	19,0	2,65	13,4
		3	18,3	2,78	11,6
		28	20,6	2,12	12,1
		31	20,6	2,79	12,1
Placebo	45037	0	23,0	2,61	11,9
		3	20,8	2,92	11,2
		28	24,1	2,14	11,0
		31	20,8	2,70	10,2
	45912	0	19.1	3,21	13,4
		3	18,6	3,72	10,3
		28	19,6	2,07	11,2
		31	20,2	4,23	11,3
	45852	0	18,4	3,02	11,6
		3	18,5	3,57	10,4
		28	18,5	2,31	11,7
		31	19,6	3,70	11,7

Table S9 – Monitoring of body weight and rectal temperature of monkeys after infection with SARS-CoV-2 virus, Delta strain

Group	Animal ID	Results of body weight (m, kg) and rectal temperature (t, ^{0}C) monitoring at days 0-6 after SAR-CoV-2 virus challenge														Δ, kg, m D0. – m D6.
		0		1		2		3		4		5		6		
		m	t	m	t	m	t	m	t	m	t	m	t	m	t	
FluCoVac-96	45914	3,90	39,4	3,95	38,6	3,91	38,0	3,93	38,6	3,96	38,8	4,00	39,0	3,98	39,1	+ 0,08
	45884	4,25	39,1	4,32	38,1	4,25	38,1	4,27	38,1	4,29	38,5	4,31	38,6	4,33	38,4	+ 0,08
	45970	4,58	39,5	4,61	39,1	4,54	38,3	4,61	38,1	4,67	38,9	4,71	38,8	4,69	39,1	+ 0,11
LAIV	45740	4,58	38,9	4,60	38,4	4,59	38,6	4,59	38,3	4,62	38,4	4,69	38,5	4.68	38,6	+ 0,10
	45841	4,00	39,5	4,02	38,6	4,07	38,7	4,10	38,6	4,15	39,1	4,17	39,2	4,08	39,1	+ 0,08
	45849	4,17	39,0	4,20	38,6	4,10	38,6	4,20	38,2	4,21	38,1	4,25	38,4	4,26	39,1	+ 0,09
Placebo	45037	4,45	39,0	4,30	38,4	4,40	38,5	4,47	38,3	4,51	38,7	4,56	38,8	4,51	38,5	+ 0,06
	45912	4,55	39,5	4,60	39,2	4,60	38,7	4,64	38,3	4,61	39,0	4,58	39,1	4,56	39,1	+ 0,01
	45852	4,42	39,3	4,35	39,0	4,41	38,7	4,47	38,6	4,50	38,9	4,54	39,0	4,50	39,2	+0,08

The normal body temperature of rhesus monkey is (38,1-39,5) ^{0}C

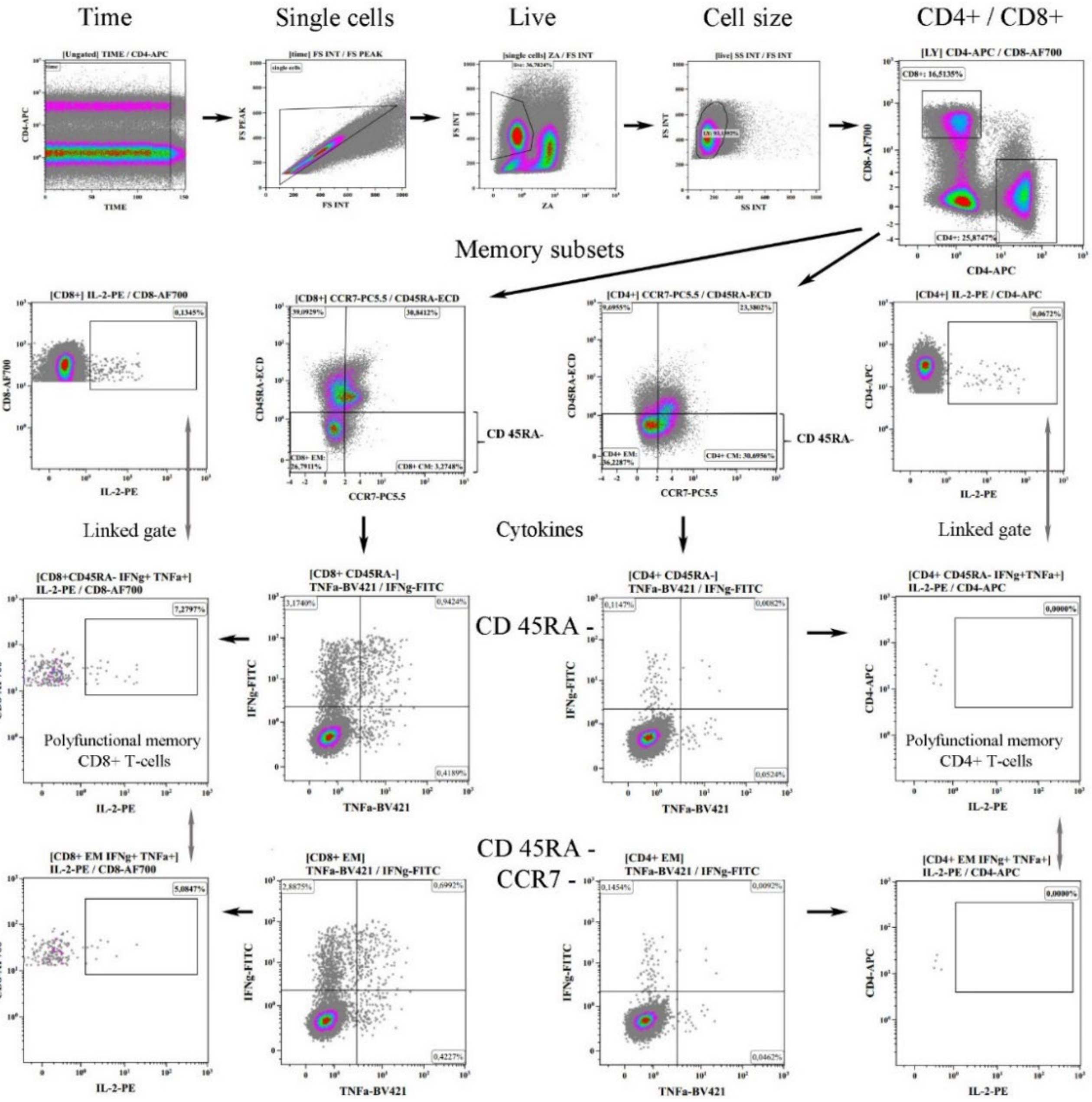

Figure S1. Gating strategy for immunophenotyping of cytokine-producing T-cells subsets in rhesus monkey PBMC. The cells were stimulated with purified live influenza virus or recombinant SARS-CoV-2 N protein overnight, than intracellular cytokine (IFNγ/TNFα/IL-2) staining was performed.

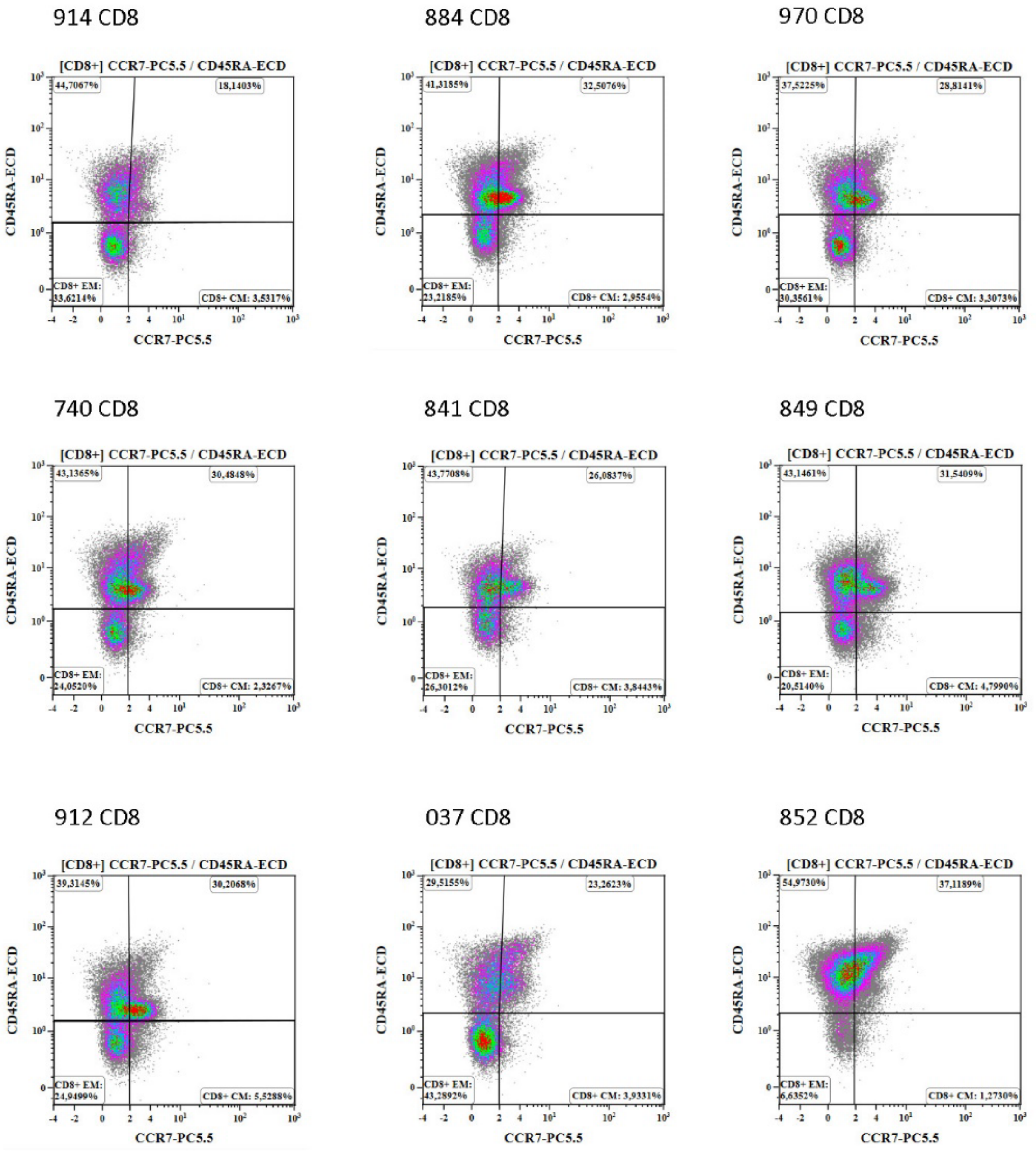

Figure S2. CD8+ memory T-cells subsets distribution in individual animals' PBMC.

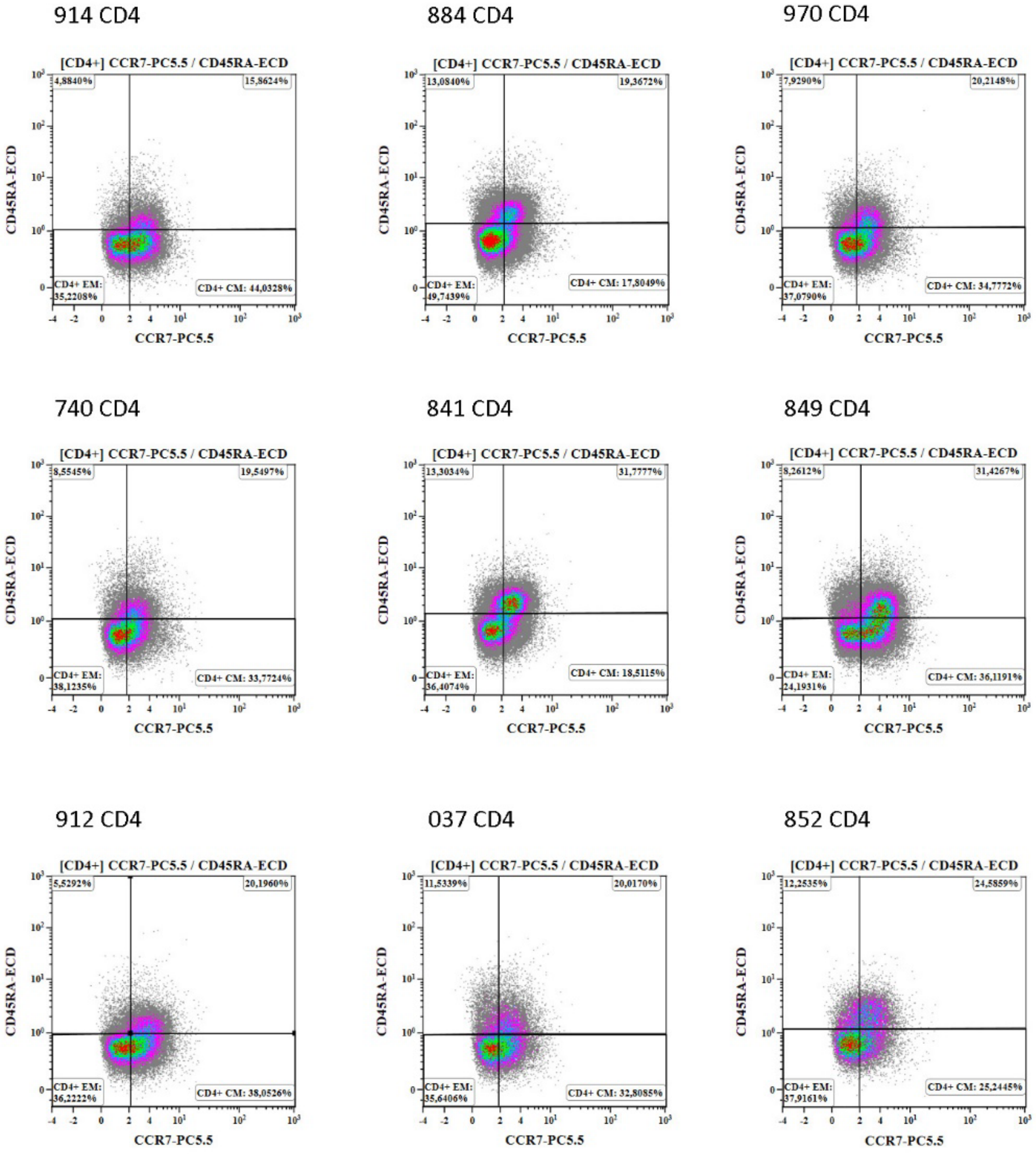

Figure S3. CD4+ memory T-cells subsets distribution in individual animals' PBMC.

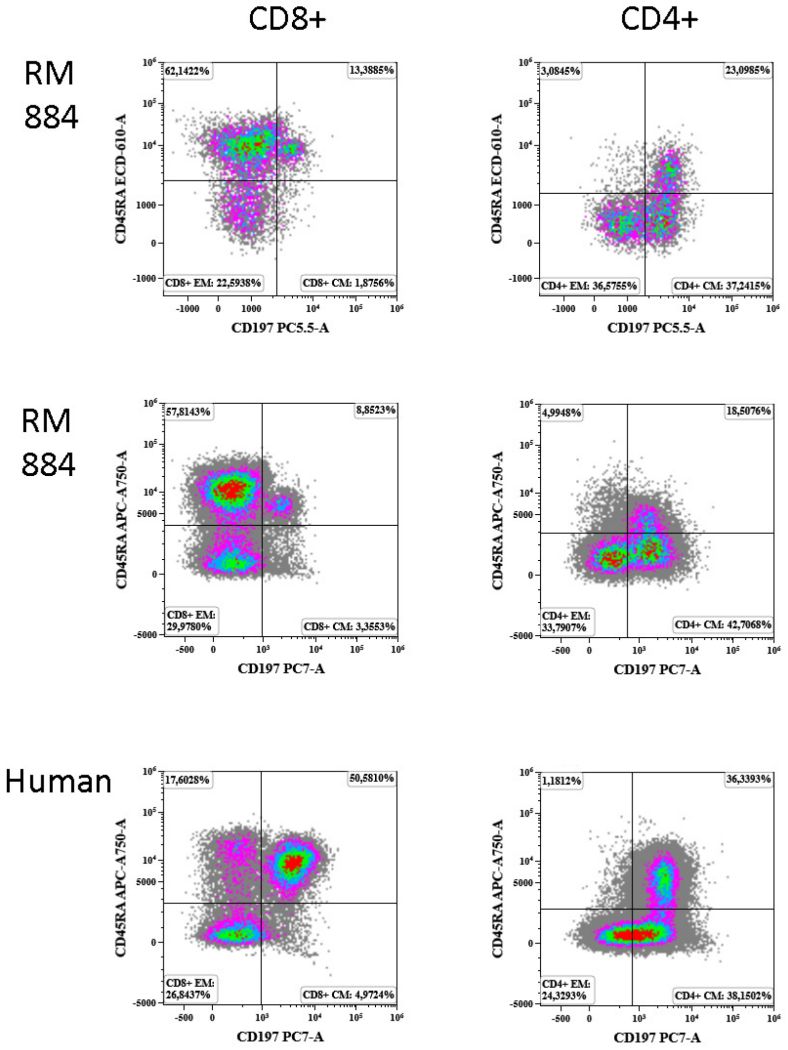

ZombieAqua Live/dead 423102 Biolegend
APC CD4 OKT4 317416 Biolegend
APC-AF700 CD8 SK1 344724 Biolegend
ECD CD45RA 2H4 im2711U Beckman Coulter
PC5.5 CD197 G043H7 353220 Biolegend

Alexa fluor 488 IFN-γ 4S.B3 502515 Biolegend
PE IL-2 MQ1-17H12 500307 Biolegend
BV421 TNF-α MAb11 502932 Biolegend

ZombieAqua Live/dead 423102 Biolegend
APC CD4 OKT4 317416 Biolegend
PerCP/Cy5.5 CD8 SK1 345774 BD
APC-AF750 CD45RA 2H4 A86050 Beckman Coulter
PE-Cy7 CD197 3D12 557648 BD

Alexa fluor 488 IFN-γ 4S.B3 502515 Biolegend
PE IL-2 MQ1-17H12 500307 Biolegend
BV421 TNF-α MAb11 502932 Biolegend

Figure S4. Memory T-cells subsets stained with different antibody panels.
To verify immunophenotyping protocol, the PBMC sample of rhesus monkey 884 was stained with two different antibody panels and memory subsets were gated. Sample of human PBMC was used as a control. The antibodies panels are listed on the figure.

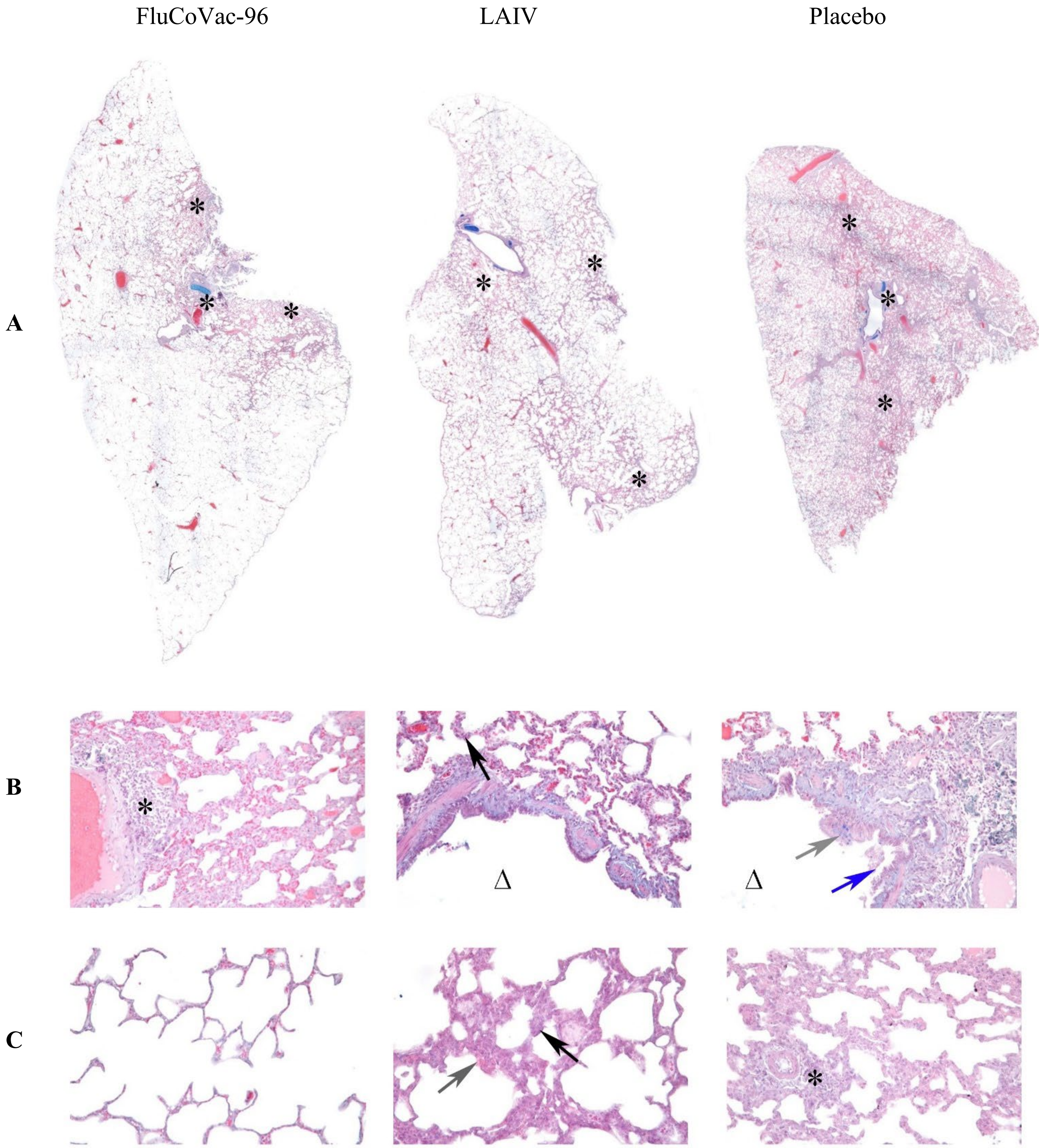

Figure S5. Representative micrographs of monkeys' lung tissue fragments.

A. Representative micrographs of lung section fragment, staining with hematoxylin-eosin, alcian blue, x40. Asterisk - areas of inflammatory consolidation of parenchyma. **B.** Representative micrographs of lung section fragment, staining with hematoxylin-eosin, alcian blue, x200.

Asterisk - mixed cellular infiltration. Triangle – lumen of terminal bronchiole. Black arrow - exfoliation of the respiratory epithelium. Gray arrow - basilar hyperplasia of the respiratory epithelium. Blue arrow - exfoliation of respiratory epithelium. **C.** Representative micrographs of lung section fragment, staining with hematoxylin-eosin, alcian blue, x200. Arrow - interalveolar septa are thickened due to edema, fulminant hemorrhage and infiltration with polymorphic leukocytes. Asterisk - mixed cellular infiltration.

In Placebo group

In a panoramic cross-sectional image, the lung tissue is unevenly airy and emphysematous. Uneven hyperemia/plethora and areas of inflammatory consolidation of the parenchyma are determined (*). The airways are passable, without pronounced changes (Figure S5 A - Placebo, magnification 40). Microscopic examination of the pulmonary parenchyma and terminal bronchiole (lumen marked *) revealed basilar hyperplasia of the respiratory epithelium (blue convoluted arrow). There is exfoliation of the respiratory epithelium (arrow). The wall of the bronchiole is edematous, infiltrated with polymorphic leukocytes. A cluster of basophils is found at the periphery (Figure S5 B - Placebo, magnification 200). An area of inflammatory consolidation of the lung tissue was identified; the interalveolar septa were thickened due to edema, congestion and infiltration with polymorphic leukocytes. A mixed cell coupling (*) is formed around the precapillary arteriole (Figure S5 C - Placebo, magnification 200).

LAIV group

Microscopic examination of the panoramic cross-sectional image revealed that the lung tissue was unevenly airy and emphysematous. Uneven hyperemia and areas of inflammatory consolidation of the parenchyma are determined (*). The airways are passable, without pronounced changes (Figure S5 A - LAIV, magnification 40). Histological examination of the pulmonary parenchyma and terminal bronchiole (lumen indicated by *) showed that the wall of the bronchiole was unchanged and the respiratory epithelium was intact. The adjacent interalveolar septa are clearly congested, edematous, infiltrated with lymphocytes, and scattered type II alveolocyte hyperplasia is determined (Figure S5 B - LAIV, magnification 200). An area of inflammatory consolidation of the lung tissue with thickening of the interalveolar septa due to edema, hyperemia and infiltration of polymorphic leukocytes (arrow) was also identified (Figure S5 C - LAIV, magnification 200).

FluCoVac-96 group

In the group with the vaccine being studied, a panoramic photograph of the lungs revealed an almost normal histological structure of the tissue. The lung tissue was markedly emphysematous, the vascular bed was uniformly full of blood, the airways and alveolar spaces were free. In the subpleural regions, compression of the interalveolar septa was noted. However, atelectasis identified due to its extensive nature mimic interstitial pneumonia (*). (Figure S5 A - FluCoVac-96, magnification 40). At a higher magnification (200-400x) of the microscope, the area of mechanical compression of the pulmonary parenchyma was studied. The interalveolar septa are normally formed and do not contain infiltration. The lumen of the alveoli is narrowed, practically undetectable (Figure S5 B - FluCoVac-96, magnification 200). In the subpleural area of emphysema, the interalveolar septa were thinned due to stretching, and the lumen of the alveoli was expanded and free (Figure S5 C - FluCoVac-96, magnification 200).
